# Supplementary material for: Eliciting preferences of persons with dementia and informal caregivers to support ageing in place in the Netherlands: a protocol for a discrete choice experiment
Source: BMJ Open. 2023 Dec 9;13(12):e075671. doi: 10.1136/bmjopen-2023-075671 (PMC10729270; doi:10.1136/bmjopen-2023-075671)
Supplement: Supplementary data [file bmjopen-2023-075671supp001.pdf]

Introduction: Thank you for participating in this study. My name is [short introduction interviewer]. The research that we are doing is focused on persons who are living at home. I would like to talk with you about your living situation at home, the daily things that you do and the help that you receive. If I ask a question that is not clear, feel free to let me know. There are no wrong answers, you can say anything that comes to mind. I would like to record the interview, if that is ok with you. The recordings will only be shared with my colleagues. The conversation will not be shared with your doctor or family. We will leave out your name. your name will be replaced by a number. *[reading information letter + informed consent – singing informed consent – start recording]*

Before we start, I have a few general questions that I would like to ask you: 1) What education did you receive? 2) Where were you born? 3) How old are you? 4) Where do you live? 5) Do you live alone? 6) Where does your family live?

Theme	Explanation theme	Examples of questions
Introduction	Start-up questions giving respondent room to talk and bring up topics.	• Can you tell something about yourself?<br>• How is it going at home?<br>• How is your health?
Daily activities	Talk about: (help with) cleaning, cooking, showering, getting dressed etc.<br><br>Find out if they get help with these activities, from who and how they value this help.	• What are daily activities that you do?<br>• What does your day look like?<br><br>• Do you receive help with these daily activities?<br>• How would you like to be helped with these activities?
Social activities	Talk about: social activities such as visiting other persons, playing cards etc.<br><br>Ask if people go to a daycare facility + how they value this.<br><br>For informal caregivers: ask if they have time for themselves, to do their own things.	• What are things that you like to do?<br>• What are things that you like to do with other people?<br><br>• With who do you like to do these activities?<br>• How would you like to be supported in this?
Adaptations in the home	Talk about: adaptations such as a stair lift, grips in the shower, alarm system etc.	• What changes are made in your house?<br>• What are things that you would like to change about your house?

Information	Talk about: information received after diagnosis.<br><br>Did they get information about what to expect or how to cope with dementia.	• Have you gotten information about what to expect (after diagnosis)?<br>• How was the support of your general practitioner/ case manager dementia?<br><br>• What questions do you have about dementia?
Cognitive training	Talk about: if people use tools to help them remember.	• How do your memory problems affect you?<br><br>• What tools do you use to help you remember?
Emotional support	Talk about: how people feel about their situation, their worries or concerns.<br><br>Do they talk about this and with who.	• How do you feel about your situation (dementia diagnosis)?<br><br>• do you have someone to discuss your concerns with?
Closing questions	Question to end the interview, ask if participants want to add something.	• What are things that you would like to get help with?<br><br>• Are there things that you would like to add?

Examples of probing questions

- With who do you do this? / Who helps you with this?
- How often do you do this? / How often do you get this help?
- What do you like about this help/support?
- What do you dislike about this (help/support)?

Thank you so much for sharing your story. *[ask if people wat to be informed about the results of the study].*
