# Supplementary material for: Eliciting preferences of persons with dementia and informal caregivers to support ageing in place in the Netherlands: a protocol for a discrete choice experiment
Source: BMJ Open. 2023 Dec 9;13(12):e075671. doi: 10.1136/bmjopen-2023-075671 (PMC10729270; doi:10.1136/bmjopen-2023-075671)
Supplement: Author's manuscript [file bmjopen-2023-075671.draft_revisions.pdf]

BMJ Open

BMJ Open is committed to open peer review. As part of this commitment we make the peer review history of every article we publish publicly available.

When an article is published we post the peer reviewers' comments and the authors' responses online. We also post the versions of the paper that were used during peer review. These are the versions that the peer review comments apply to.

The versions of the paper that follow are the versions that were submitted during the peer review process. They are not the versions of record or the final published versions. They should not be cited or distributed as the published version of this manuscript.

BMJ Open is an open access journal and the full, final, typeset and author-corrected version of record of the manuscript is available on our site with no access controls, subscription charges or pay-per-view fees (http://bmjopen.bmj.com).

If you have any questions on BMJ Open's open peer review process please email info.bmjopen@bmj.com

BMJ Open

Eliciting preferences of persons with dementia and informal caregivers to support ageing in place in the Netherlands: A protocol for a discrete choice experiment.

Journal:	*BMJ Open*
Manuscript ID	bmjopen-2023-075671
Article Type:	Protocol
Date Submitted by the Author:	16-May-2023

Complete List of Authors:	Vullings, Isabelle; Amsterdam UMC Locatie AMC, Geriatrics; Amsterdam Public Health Research Institute<br>Uysal-Bozkir, Özgül; Erasmus University Rotterdam Erasmus School of Social and Behavioural Sciences, Department of Psychology, Education and Child Studies<br>Smits, Carolien; Pharos center of expertise on Health disparities<br>Wammes, Joost; Amsterdam UMC Locatie AMC, Geriatrics; Amsterdam Public Health Research Institute<br>Labrie, Nanon ; Vrije Universiteit Amsterdam, department of language, literature & communication<br>Swait, J.D.; Erasmus University Rotterdam, Erasmus Choice Modelling Centre; Erasmus University Rotterdam, Erasmus School of Health Policy & Management<br>de Bekker-Grob, Esther; Erasmus University Rotterdam, Erasmus Choice Modelling Centre; Erasmus University Rotterdam, Erasmus School of Health Policy & Management<br>Macneil-Vroomen , Janet; Amsterdam Universitair Medische Centra, Geriatrics ; Amsterdam Public Health Research Institute

Keywords:	Dementia, Patient Participation, Decision Making

SCHOLARONE™
Manuscripts

Eliciting preferences of persons with dementia and informal caregivers to support ageing in place in the Netherlands: A protocol for a discrete choice experiment.

Isabelle Vullings[1-2], Özgül Uysal-Bozkir[3], Carolien Smits[4], Joost Wammes[1-2], Nanon Labrie [5], Joffre Swait [6-7], Esther de Bekker-Grob[6-7], Janet L. MacNeil Vroomen [1-2]

1. Department of Internal Medicine, Section of Geriatric Medicine, Amsterdam UMC, Academic Medical Center, University of Amsterdam, Amsterdam, The Netherlands.
2. Amsterdam Public Health research institute, Aging & Later Life, Amsterdam, The Netherlands
3. Erasmus School of Social and Behavioural Sciences, Department of Psychology, Education and Child Studies, Erasmus University Rotterdam, Netherlands.
4. Pharos Center of Expertise on Health Disparities, Utrecht, The Netherlands
5. Department of Language, Literature & Communication, Vrije Universiteit Amsterdam, Amsterdam, The Netherlands
6. Erasmus School of Health Policy & Management, Erasmus University Rotterdam, Rotterdam, The Netherlands.
7. Erasmus Choice Modelling Centre, Erasmus University Rotterdam, Rotterdam, The Netherlands.

Corresponding author:
Msc Isabelle Vullings, i.vullings@amsterdamumc.nl
- Department of Internal Medicine, Section of Geriatric Medicine, Amsterdam UMC, Academic Medical Center, University of Amsterdam, The Netherlands.
- Amsterdam Public Health research institute, Aging & later life, Amsterdam, The Netherlands.

Keywords: dementia, patient participation, decision making, discrete choice experiment, ageing in place, informal caregivers

Word count: 3925

For peer review only

ABSTRACT

Introduction: Ageing in place (AIP) for persons with dementia is encouraged by European governments and societies. Healthcare packages may need reassessment to account for the preferences of care funders, patients, and informal caregivers. By providing insight into people's preferences, Discrete Choice Experiments (DCEs) can help develop consensus between stakeholders. This protocol paper outlines the development of a Dutch national study to co-create a healthcare package design methodology built on DCEs that is person-centered and helps support informal caregivers and persons with dementia to AIP. A subpopulation analysis of persons with dementia with a migration background is planned due to their high risk for dementia and underrepresentation in research and care.

Methods and analysis: The DCE is designed to understand how persons with dementia and informal caregivers choose between different healthcare packages. Qualitative methods are used to identify and prioritize important care components for persons with dementia to AIP. This will provide a list of care components that will be included in the DCE, to quantify the care needs and preferences of persons with dementia and informal caregivers. The DCE will identify individual and joint preferences to AIP. The relative importance of each attribute will be calculated. The DCE data will be analysed with the use of a random parameters logit (RPL) model.

Ethics and dissemination: Ethics approval was waived by the Amsterdam University Medical Center (W23_112 #23.137). A study summary will be available on the websites of Alzheimer Nederland, Pharos, and Amsterdam Public Health institute. Results are expected to be presented at [inter]national conferences, peer-reviewed papers will be submitted, and a dissemination meeting will be held to bring stakeholders together. The study results will help improve healthcare package design for all stakeholders.

STRENGTS AND LIMITATIONS OF THE STUDY

- This will be the first study to include informal caregivers and persons with dementia to identify ageing in place preferences that has been co-designed with health insurers, policy makers, patient advocacy groups, healthcare professionals, researchers, informal caregivers, and persons with dementia.
- Innovative and rigorous economic methods will be employed to evaluate ageing in place preferences.
- Pooling of persons with dementia with different migration background may lead to better understanding of their needs as they are typically underrepresented in research.
- A limitation of this study is the generalizability of results as not all migrant groups living in the Netherlands may be included.
- This study is conducted in the Netherlands, findings might be specific to the Dutch Healthcare setting.

INTRODUCTION

Nearly every country across Europe has reformed its long-term care policy to emphasize ageing in place as a way to control costs associated with population ageing[1]. As the highest spender of long-term care per gross domestic product in the world[2], the Netherlands also reformed its long-term care policy in 2015. The reform increased informal care duties and encouraged older adults to age in place [3 4]. A

substantial component of the Dutch long-term care costs is attributed to dementia care as it is the most costly disease encompassing 10.6% of the total Dutch healthcare budget[5]. The dementia population is known for its requirement of complex and costly care[6]. However, it remains unknown how ageing in place has affected this population. In the years after the reform, preliminary results indicate an increased need for crisis help, unplanned hospitalizations, acute nursing home admissions, and hospital deaths[7 8]. These crises situations are not only costly, but also raise questions about the safety and well-being of persons with dementia ageing in place, in addition to the informal caregivers expected to carry the load.

Despite the need for evidenced-based policy to ensure proper healthcare services, people with dementia and informal caregivers are frequently excluded from policy creation; therefore, services often do not fit with their needs or are not provided at the right time [9 10]. To support successful ageing in place, it is crucial to identify the care preferences of persons with dementia and informal caregivers. Whilst identifying these preferences, special attention should be given to persons with dementia and informal caregivers with a migration background, who are known to have a higher risk of developing dementia[11 12]. Despite their high risk of developing dementia, person with a migration background are underrepresented in research and care [13]. It is crucial that persons with a migration background be included in research so healthcare package evidence is representative of the dementia population. To provide recommendations for healthcare packages it is subsequently needed to quantify the identified care preferences, which can be realized with the use of a discrete choice experiment.

Discrete choice experiments (DCEs) are increasingly used as a quantitative method to determine patients' preferences in healthcare [14 15]. In a DCE participants are presented with a series of alternative hypothetical scenarios. In the DCE participants are repetitively asked to select in each alternative hypothetical scenario their preferred option from amongst a presented set of options, e.g., care packages [15]. These choice options are characterized by their attributes and corresponding attribute levels [14 15]. An example of an attribute could be emotional support, described as the possibility to talk to someone about personal feelings or concerns. The corresponding attribute levels could be (1) a peer support group, (2) a psychologist or (3) a telephone helpline. The outcomes (choices) from DCEs are analyzed based on an assumption that participants act in a utility maximizing manner, choosing their most preferred option based on the relative overall attractiveness of the included attributes and attribute levels [15 16]. This protocol paper will outline the development of a Dutch national study to create a healthcare package design methodology, built on DCEs, that is person-centered and helps support both informal caregivers and persons with dementia to successfully age in place. Qualitative methods will be used to identify the care needs and preferences of persons with dementia and their informal caregivers. The results of the qualitative studies will provide insights into the attributes (i.e., care components) that will be included in the DCE. For the DCE, a subpopulation analysis of persons with dementia with a migration background is planned.

Aims

The aims of this study are to identify individual and joint preferences of persons with dementia and informal caregivers for in-home support that enables ageing in place. We also aim to create optimal healthcare packages for persons with dementia and informal caregivers to age in place. For this second aim, choice model inferences will be the basis for creating the most preferred care packages and testing the uptake in a subsample of participants. This will validate the national model inferences and provide policymakers high quality, understandable and implementable evidence.

METHODS

Overview of the discrete choice experiment

For peer review only - http://bmjopen.bmj.com/site/about/guidelines.xhtml

In this protocol paper, the different phases of designing an inclusive DCE study in which persons with dementia and their informal caregivers can participate will be outlined. Figure 1 illustrates that the first phase is focused on attribute development, in which semi-structured interviews are used to identify important components of care for persons with dementia and informal caregivers ageing in place. In the second phase persons with dementia and informal caregivers are asked which of the care components are most important to them, leading to a prioritization of the attributes and the development of attribute levels. For phases one and two, the COREQ guidelines for reporting qualitative research will be followed [17]. The third phase consists of a pilot which is used to test if the content of the DCE is understandable for persons with dementia and informal caregivers, and if the list of included attributes and corresponding attribute levels is complete. Finally, in phase four the researchers conduct the DCE study. Figure 1 shows that an expert panel will be consulted during all the different phases of the study to reflect upon the research design and findings. Phases three and four will be reported according to the STROBE guidelines for observational studies[18] and for constructing the DCE design the ISPOR good research practices reports will also be followed [19 20].

Patient and public involvement

The DCE, including the framing of the questions, will be developed after focus group discussion and multiple individual interviews with persons with dementia and informal caregivers. Persons with dementia, informal caregivers and policymakers are involved from the start of the study to ensure the appropriateness of the methodology and to help come to meaningful and implementable results [21]. Patient advocacy groups, persons with dementia and informal caregivers are to be included to make sure that the study properly represents these groups, and the study results reflect their needs and preferences. We will also follow and report this involvement using the GRIPP2 checklist [22]. The DCE is designed to be inclusive and has a manageable cognitive load for the participants [21]. The DCE study will identify individual and joint preferences to AIP, allowing for this concept to be implemented elsewhere. The findings of this study will be published in peer-reviewed journals. An easy-to-understand summary of the results will be available through organizations such as Alzheimer Nederland, Pharos, and the Amsterdam Public Health research institute. A policy brief will be written to ensure that the study results are known to policy creators and can help to improve healthcare package design for older adults and informal caregivers.

Expert panel

Throughout the entire study, an expert panel will be consulted on the design and content of the DCE, and on the inferences made from its execution. The expert panel will include researchers who have experience with doing DCEs, policy makers, health insurers, patient advocacy groups, persons with dementia and informal caregivers. The experienced researchers will be asked to provide guidance and feedback on the methodological choices made in the DCE design. Subsequently, the envisioned outcome of the DCE study will be discussed with policymakers and health insurers to gain insights concerning the comprehension and appropriateness of this envisioned outcome. The inclusion of policymakers and health insurers in this phase will allow for the opportunity to make changes to the study design that help to realize implementation.

Attribute and level development for Dutch persons with dementia and informal caregivers

Study phases one and two are already partly completed, as interviews and focus groups were held with persons with dementia and their informal caregivers across the Netherlands to identify attributes and levels (Table one) [23 24]. We found that informal caregivers and people with dementia had different care priorities [24]. People with dementia prioritized day-to-day help and social care, while for informal caregivers, priorities were information about dementia, organisation of care, and emotional support[24].

For peer review only - http://bmjopen.bmj.com/site/about/guidelines.xhtml

Unfortunately, no persons with a migration background were included in the previous qualitative studies. Therefore, the interviews and focus groups will be repeated with persons with dementia and informal caregivers with a migration background to ensure the attributes and levels are applicable to this part of society as they have a higher risk for dementia compared to people with non-migration background[11].

Table 1. Attribute description and corresponding levels

Attribute	Description	Possible levels
In-home care	I can get assistance at home with personal care such as showering, dressing, or medication	- Daily on a fixed time<br>- 24/7 on demand
Help with daily activities	I can get assistance at home with household tasks such as groceries, laundry, cooking, cleaning, or help with doing my finances	- Once per week<br>- Multiple times per week
Social activities	I can participate in social activities that I like to do	- At a day-care facility<br>- At home
Emotional support	I can talk to someone when I feel down or want to share my worries	- Peer support group<br>- Psychologist<br>- Telephone helpline<br>- Case manager
Information about dementia	I can get information about having dementia	- Telephone helpline<br>- Case manager
Navigating the healthcare system	I can get assistance with organizing care, and help with insurances	- Telephone helpline<br>- Case manager
Home adaptations and tools	I can get home adaptations and tools such as a stair lift, grips in shower and toilet, or a personal alarm	- No reimbursements<br>- Fully reimbursed

Note: current list of attributes may need to be updated after expert panel review and interviews with persons with dementia and informal caregivers with a migration background

Attribute identification for persons with dementia and informal caregivers with migration background

To identify care preferences and needs of persons with dementia and informal caregivers with a migration background, semi-structured interviews will be performed across the Netherlands. Participants will include persons with dementia and informal caregivers with a Turkish, Moroccan, or Surinamese background as these are the largest groups of non-western immigrants in the Netherlands and they are known to have a high risk for developing dementia[11]. People living with dementia who do not speak Dutch as their first language will also be welcome to participate. Participants will be recruited with the help of day-care centers and organizations that serve persons with a migration background (Pharos and NOOM). The organizations will inform potential participants about the study, and interested potential participants will be provided with a bilingual invitation letter, in which the study will be further explained. The interviews will be conducted in the preferred language of the participants: Turkish, Moroccan-Arabic, Tarifit, or Dutch[11]. Prior to the interviews, participants will provide written informed consent and it will be stressed that participation can be stopped at any moment. Interviews will be conducted by trained bilingual interviewers. Persons with dementia and informal caregivers will be interviewed separately. However, since it is important that participants feel comfortable, if they strongly wish to be interviewed together this will be permitted.

The interviews will be semi-structured, which means that an interview guide will be used to provide some structure, whilst leaving room for new topics to be brought up by the participants. The interview guide will generally be based on the guide of the interview study used previously for persons with non-

For peer review only

migration backgrounds [23], with additions based on findings of previous studies that focused on persons with dementia and informal caregivers with a migration background [25 26]. The cultural sensitivity and understandability of the interview guide will be assessed by a knowledge institute and experienced researchers (CS and ÖUB). The interviews will be transcribed and translated by the bilingual interviewer who conducted the interviews. Respondents will have the opportunity to review the translated transcripts, which will help to ensure proper interpretation and provide the respondents with the opportunity to, if necessary, give clarifications. It will be stressed that this is voluntary, and that participants are free to choose if they wish to read the translated transcripts. Interviews will be held until data saturation is reached, which is expected after approximately 10 interviews for each group [27]. Thematic analysis will be used to analyze and identify relevant patterns within the data [28]. Coding of the data will be done with both a deductive and inductive approach, starting with certain theoretical or empirical assumptions, with an open approach [28]. The results will provide a list of attributes (care components) that are important for persons with dementia and informal caregivers with a migration background.

Attribute prioritization and the development of attribute levels

To understand the relative importance of the care components identified, mixed focus group sessions will be held with a new sample of persons with dementia and informal caregivers with a Turkish, Moroccan, or Surinamese background[11]. Mixed focus groups will create an environment in which participants can share their experiences and build on each other's knowledge [24]. To ensure a free discussion, informal caregivers will not be participating in the same focus group session as their person with dementia [24]. Participants will be recruited with the help of the previously mentioned organizations. To facilitate dialogue between the participants, focus groups will include participants with similar migration backgrounds and linguistic preferences. The focus groups will be conducted by trained bicultural interviewers. The focus group session will include a quantitative ranking exercise in which persons with dementia and informal caregivers will be asked to list the care components from most important to least important [24]. For this ranking exercise, the research team will use cards with the previously identified care characteristics, containing visual and written information. During the focus group meetings, the reasoning behind the choices made in the ranking exercise will be discussed. Focus group sessions will be held until saturation is reached, which is expected after approximately six sessions, each including persons with dementia (n=4) and informal caregivers (n=4). Focus groups will be transcribed and translated by the trained bilingual interviewers. Participants will be provided with the opportunity to read the translated transcripts and, if necessary, provide clarifications. Thematic analysis will be used to analyze and identify relevant patterns within the data [28]. Coding of the data will be done with both a deductive and inductive approach, starting with certain theoretical or empirical assumptions, with an open approach [28]. The results will consist of a ranking of the attributes (care components) and a description of the attribute levels (characteristics of care components).

Discrete choice experiment pilot

For the DCE pilot the list of attributes that has previously been identified (Table 1) will be updated with the findings of the qualitative studies with persons with dementia and informal caregivers with a migration background. The pilot will include both individual DCE rounds with the persons with dementia (n=4) and informal caregivers (n=4), and dyadic rounds with both the person with dementia and their informal caregiver. The in-person pilot will include the testing of respondents' understanding of choice tasks and testing of the appropriateness and understanding of the included attributes and attribute levels. Finally, persons with dementia will be asked to think aloud whilst completing the choice tasks. The think-aloud technique is commonly used in research to make thought processes of the participants observable for the researchers [29]. It has been found particularly useful in supporting

For peer review only

persons with dementia to complete DCE choice tasks, as it enables researchers to observe difficulties and, if necessary, provide extra guidance or remind persons with dementia about the rules of the choice tasks [30]. Furthermore, the pilot will help to illustrate if the length of the DCE survey is acceptable and how long participants need to complete the choice tasks. Based on the pilot, changes might need to be made to the DCE survey to increase appropriateness and understandability, as well as manage survey complexity for persons with dementia.

The final discrete choice experiment

The final DCE will include three rounds: (1) persons with dementia, (2) informal caregivers, and (3) dyadic rounds with both the persons with dementia and informal caregivers. The first two rounds will help to elucidate individual preferences, whilst the dyadic round will help to elucidate joint preferences. Identifying joint preferences is important since decisions about care are often made by multiple individuals [31-33]. Furthermore, the dyadic DCE round can pose as an opportunity for the person with dementia and their informal caregiver to have an in-depth conversation about their preferences and needs, which could lead to better mutual understanding [30]. A recent study indicated that informal caregivers were not per se dominant in dyadic DCE rounds, in fact the informal caregivers were found to be helpful in assisting the person with dementia to complete more choice tasks [30].

The complexity of a DCE can be an overwhelming challenge for persons with dementia [34-36]. The assistance of the informal caregiver can therefore be of great help for the dyadic DCE rounds [30]. In addition, there are a few other options in the DCE design that will help to lower the cognitive burden for person with dementia. First, illustrations will be used to visualize the choice sets and included attributes. Figure 2 provides an example of such an illustration [30]. The illustrations are made by a graphic designer who has experience illustrating pictures for persons with cognitive impairments. Second, the DCE will have binary choice tasks, asking participants to choose between packages A and B, instead of including multiple alternatives (e.g., additional packages C or D).

Power calculation

Envisioning a two-alternative choice task and four replicates per dyad (person with dementia and informal caregiver), it is recommended to have a minimum sample size n≥576 for a discrete choice experiment to reliably achieve 95% confidence intervals on true uptake probabilities of 0.4 [37]. Therefore, the goal will be to achieve a sample size of around 600 dyads of persons with dementia and informal caregivers, covering all targeted groups.

Purposive sampling of minority groups, people living in urban and rural areas, and people with different educational levels will be used. In addition, sampling across the country will help to make the healthcare package as nationally representative as possible [38]. Participants will be recruited with the help of organizations that serve persons with dementia and their informal caregivers.

Outcomes

The main outcome of the DCE study will be the relative importance of the attributes (care components) included in the DCE choice tasks. The relative importance of each attribute will be calculated as the difference between the preference weights of the most and least preferred level of that attribute [39]. The relative importance will be scaled so that 10 indicates the most preferred attribute.

A contingent valuation approach will be used to identify the participants willingness to pay for the most preferred programs [40 41]. If participants indicate that they would prefer the envisioned package

For peer review only

over their current service provision, they will be asked if they are willing to pay an additional monthly out of pocket fee of €50 for this package. If participants answer this question with yes, additional questions with out of pocket fees of €100 and €200 will be asked [40]. If participants answered no to the initial question of €50, similar questions with an amount of €25 and €12,50 will be asked [40]. Finally, participants will be asked to indicated their maximum willingness to pay in an open-ended question [40]. For the analysis, the maximum price expressed in the open-ended question will be used and summarized through the median and interquartile range [40].

Statistical analysis

The DCE data will be analysed with the use of a random parameters logit (RPL) model [39]. For each attribute level, coefficient estimates will be estimated from the RPL model, which can be interpreted as the relative preference weight [39]. Individual and joint models will be created to evaluate preferences of persons with dementia and informal caregivers at the individual and joint DCE rounds.

To analyse if persons with dementia and informal caregivers with a migration background have different preferences, post hoc subgroup analysis will be conducted. Dummy coded variables will be added to the RPL model to identify participants that are part of given subgroups. The attribute levels in the RPL model will be interacted with the dummy variable, and all interaction terms will be added to the original RPL model [39]. The estimated parameters of the interaction terms will illustrate the difference in preferences between the subgroup (persons with a migration background) and the reference group (persons without a migration background) [39]. This method can also be used to analyse if there are different preferences based on other subgroups, such as socio-economic status.

Finally, the results of the attributes with the highest relative importance can be used to build most preferred programs (healthcare packages). To ensure the validity of these results for health insurers and the government, the uptake of the most preferred programs will be tested on a subsample of the target population. Model predictions will be verified by creating the most preferred programs according to the preferences of persons with dementia and informal caregivers that were found in the DCE. A new sample will be collected of 50-60 participants to test the uptake of the most preferred programs by asking participants which one they would rather have over their current service provision [42].

DISCUSSION

The discrete choice methodology described in this paper enables persons with dementia and informal caregivers to participate in policy formulation and evaluation. The use of this methodology enables care based on the needs of persons with dementia and their informal caregivers. It is assumed that this will help to avoid crisis situations and improve the quality of life of persons with dementia and their informal caregivers. Furthermore, among the outcome of the final DCE study will be a ranking of the most preferred care components represented in a care package. This helps health insurers and the ministry of health to maximize their chances for designing healthcare packages that are appropriate, useful, and meaningful.

Furthermore, this study protocol provides an important methodological contribution to the academic field. It provides a clear guide on how to build an inclusive DCE study, covering the important methodological choices throughout all the required study phases. Moreover, it provides guidelines on how to include underrepresented groups in DCE studies. The methodological choices described to help include persons with dementia with a migration background, can also be used to include other underrepresented groups such as people with low literacy or people with low health literacy [43 44]. Finally, this study provides an example of how to overcome language barriers in research and knowledge dissemination.

For peer review only

Strengths and limitations of this study

A strength of this study will be that it is the first study to include informal caregivers and persons with dementia to identify ageing in place preferences that has been co-designed with health insurers, policy makers, patient advocacy groups, healthcare professionals, researchers, informal caregivers, and persons with dementia. Innovative and rigorous economic methods will be employed to evaluate ageing in place preferences. Another strength is the inclusion of persons with dementia with different migration backgrounds, which can lead to a better understanding of their needs since they are typically underrepresented in research. A limitation of this study the generalizability of results, as not all migrant groups living in the Netherlands may be included; however, we will strive through our recruitment plan to make this study as inclusive as possible.

Ethics and dissemination

Based on the study protocol, the Ethics Committee (METC) of the Amsterdam University Medical Centre waived the obligation for the study to undergo formal ethical approval as is described under Dutch law in the Medical Research in Humans Act, January 2019 (ref W23_112 #23.137).

Consent

As this is a prospective study and pseudonymised data is used, written informed consent will be obtained from the participants prior to participation. This is consistent with current European legislation under the General Data Protection Regulation (GDPR). This study will abide by the Declaration of Helsinki and present ethical requirements.

Authors' contributions

JMV conceptualized the study and developed the study design together with the other researchers. IV and JW contributed to the development of the study design, data collection and analysis. The authors provided input for the study design from their own area of expertise; inclusive research (CS & ÖUB), qualitative research (NL), discrete choice experiments (EBG & JS). IV wrote the manuscript, the co-authors read, edited, and approved the final version.

Funding statement

This work was supported by Alzheimer Nederland, (WE,06-2021-04). In addition, grant support was from The Netherlands Organisation for Scientific Research (https://www.nwo.nl/en) (NWO-Talent-Scheme-Vidi-Grant No. 09150171910002) to EBG.

Competing interest statement

No competing interests to report.

References

1. Spasova S, Baeten, R., Coster, S., Ghailani, D., Peña-Casas, R. and Vanhercke, B. Challenges in long-term care in Europe. A study of national policies. European Social Policy Network (ESPN). Brussels: European Commission, 2018.
2. Organisation for Economic Cooperation and Development. long-term care spendings and unit costs Health at a Glance 2021. Paris: OECD publishing 2021.

For peer review only

3. Alders P, Schut FT. The 2015 long-term care reform in the Netherlands: Getting the financial incentives right? *Health Policy* 2019;123(3):312-16.
4. Maarse JH, Jeurissen PP. The policy and politics of the 2015 long-term care reform in the Netherlands. *Health Policy* 2016;120(3):241-45.
5. RIVM. Effecten van vergrijzing steeds beter zichtbaar in uitgaven gezondheidszorg 2022 [Available from: https://www.rivm.nl/nieuws/effecten-van-vergrijzing-steeds-beter-zichtbaar-in-uitgaven-gezondheidszorg#:~:text=In%202019%20werd%20in%20Nederland,naar%20de%20kosten%20van%20ziekten.
6. Wimo A, Guerchet M, Ali G-C, et al. The worldwide costs of dementia 2015 and comparisons with 2010. *Alzheimer's & Dementia* 2017;13(1):1-7.
7. Francke A, Heide I, Bruin Sd, et al. Een samenhangend beeld van dementie en dementiezorg: kerncijfers, behoeften, aanbod en impact. Themarapportage van de Staat van Volksgezondheid en Zorg. 2018
8. MacNeil-Vroomen JL, van der Steen JT, Holman R, et al. Hospital deaths increased after reforms regardless of dementia status: an interrupted time-series analysis. *Journal of the American Medical Directors Association* 2021;22(7):1507-11.
9. Van der Roest HG, Meiland FJ, Comijs HC, et al. What do community-dwelling people with dementia need? A survey of those who are known to care and welfare services. *International psychogeriatrics* 2009;21(5):949-65.
10. Van Der Roest HG, Meiland FJ, Maroccini R, et al. Subjective needs of people with dementia: a review of the literature. *International Psychogeriatrics* 2007;19(3):559-92.
11. Parlevliet JL, Uysal-Bozkir Ö, Goudsmit M, et al. Prevalence of mild cognitive impairment and dementia in older non-western immigrants in the Netherlands: a cross-sectional study. *Int J Geriatr Psychiatry* 2016;31(9):1040-9. doi: 10.1002/gps.4417 [published Online First: 2016/01/23]
12. Selten J-P, Termorshuizen F, van Sonsbeek M, et al. Migration and dementia: a meta-analysis of epidemiological studies in Europe. *Psychological Medicine* 2021;51(11):1838-45.
13. Schmachtenberg T, Monsees J, Hoffmann W, et al. Comparing national dementia plans and strategies in Europe–is there a focus of care for people with dementia from a migration background? *BMC Public Health* 2020;20:1-10.
14. de Bekker-Grob EW, Ryan M, Gerard K. Discrete choice experiments in health economics: a review of the literature. *Health economics* 2012;21(2):145-72.
15. Soekhai V, de Bekker-Grob EW, Ellis AR, et al. Discrete choice experiments in health economics: past, present and future. *Pharmacoeconomics* 2019;37(2):201-26.
16. Clark MD, Determann D, Petrou S, et al. Discrete choice experiments in health economics: a review of the literature. *Pharmacoeconomics* 2014;32(9):883-902.
17. Tong A, Sainsbury P, Craig J. Consolidated criteria for reporting qualitative research (COREQ): a 32-item checklist for interviews and focus groups. *International journal for quality in health care* 2007;19(6):349-57.
18. Cuschieri S. The STROBE guidelines. *Saudi J Anaesth* 2019;13(Suppl 1):S31-s34. doi: 10.4103/sja.SJA_543_18 [published Online First: 2019/04/02]
19. Johnson FR, Lancsar E, Marshall D, et al. Constructing experimental designs for discrete-choice experiments: report of the ISPOR conjoint analysis experimental design good research practices task force. *Value in health* 2013;16(1):3-13.
20. Bridges JF, de Bekker-Grob EW, Hauber B, et al. A roadmap for increasing the usefulness and impact of patient-preference studies in decision making in health: A good practices report of an ISPOR task force. *Value in Health* 2023;26(2):153-62.
21. Illes J, Lawson A, McDonald PJ. Ethical Considerations for Discrete Choice Experiments with Caregivers. *Journal of Empirical Research on Human Research Ethics* 2022;17(4):426-30.
22. Staniszewska S, Brett J, Simera I, et al. GRIPP2 reporting checklists: tools to improve reporting of patient and public involvement in research. *BMJ* 2017;358:j3453. doi: 10.1136/bmj.j3453

For peer review only

23. Vullings I, Labrie N, Wammes JD, et al. Important components for Dutch in-home care based on qualitative interviews with persons with dementia and informal caregivers. *Health Expectations* 2020;23(6):1412-19.
24. Wammes JD, Labrie NH, Agogo GO, et al. Persons with dementia and informal caregivers prioritizing care: A mixed-methods study. *Alzheimer's & Dementia: Translational Research & Clinical Interventions* 2021;7(1):e12193.
25. Lillekroken D, Halvorsrud L, Gulestø R, et al. Family caregivers' experiences of providing care for family members from minority ethnic groups living with dementia: A qualitative systematic review. *Journal of Clinical Nursing* 2021
26. Duran-Kiraç G, Uysal-Bozkir Ö, Uittenbroek R, et al. Accessibility of health care experienced by persons with dementia from ethnic minority groups and formal and informal caregivers: A scoping review of European literature. *Dementia* 2022;21(2):677-700.
27. Hennink MM, Kaiser BN, Marconi VC. Code saturation versus meaning saturation: how many interviews are enough? *Qualitative health research* 2017;27(4):591-608.
28. Braun V, Clarke V. Using thematic analysis in psychology. *Qualitative research in psychology* 2006;3(2):77-101.
29. Uysal-Bozkir Ö, Fokkema T, MacNeil-Vroomen JL, et al. Translation and validation of the De Jong Gierveld loneliness scale among older migrants living in the Netherlands. *The Journals of Gerontology: Series B* 2017;72(1):109-19.
30. Wammes JD, Swait JD, de Bekker-Grob EW, et al. Dyadic Discrete Choice Experiments Enable Persons with Dementia and Informal Caregivers to Participate in Health Care Decision Making: A Mixed Methods Study. *Journal of Alzheimer's Disease* 2022(Preprint):1-10.
31. Corfman KP, Lehmann DR. Models of cooperative group decision-making and relative influence: An experimental investigation of family purchase decisions. *Journal of Consumer Research* 1987;14(1):1-13.
32. Hensher DA, Beck MJ, Rose JM. Accounting for preference and scale heterogeneity in establishing whether it matters who is interviewed to reveal household automobile purchase preferences. *Environmental and Resource Economics* 2011;49(1):1-22.
33. Groen-van de Ven L, Smits C, Span M, et al. The challenges of shared decision making in dementia care networks. *International Psychogeriatrics* 2018;30(6):843-57.
34. Chester H, Clarkson P, Davies L, et al. People with dementia and carer preferences for home support services in early-stage dementia. *Aging & Mental Health* 2018;22(2):270-79.
35. O'Philbin L, Woods B, Holmes E. People with dementia and caregiver preferences for digital life story work service interventions. A discrete choice experiment and digital survey. *Aging & Mental Health* 2020;24(2):353-61.
36. Ratcliffe J, Hutchinson C, Milte R, et al. How do people with dementia and family carers value dementia-specific quality of life states? An explorative "Think Aloud" study. *Australasian Journal on Ageing* 2019;38:75-82.
37. Louviere JJ, Hensher DA, Swait JD. Stated choice methods: analysis and applications: Cambridge university press 2000.
38. Valliant R, Dever JA, Kreuter F. Practical tools for designing and weighting survey samples: Springer 2013.
39. Rosenow F, Winter Y, Leunikava I, et al. Relative importance of clinical outcomes and safety risks of antiseizure medication monotherapy for patients and physicians: Discrete choice experiment eliciting preferences in real-world study" VOTE". *Epilepsia* 2022;63(2):451-62.
40. Vo NX, Nguyen TTH, Van Nguyen P, et al. Using contingent valuation method to estimate adults' willingness to pay for a future coronavirus 2019 vaccination. *Value in Health Regional Issues* 2021;24:240-46.
41. Basu R. Willingness-to-pay to prevent Alzheimer's disease: a contingent valuation approach. *International journal of health care finance and economics* 2013;13(3):233-45.

For peer review only

42. Janssen EM, Marshall DA, Hauber AB, et al. Improving the quality of discrete-choice experiments in health: how can we assess validity and reliability? *Expert review of pharmacoeconomics & outcomes research* 2017;17(6):531-42.
43. Hughson J-a, Woodward-Kron R, Parker A, et al. A review of approaches to improve participation of culturally and linguistically diverse populations in clinical trials. *Trials* 2016;17(1):1-10.
44. Woodward-Kron R, Hughson J-a, Parker A, et al. Culturally and linguistically diverse populations in medical research: perceptions and experiences of older Italians, their families, ethics administrators and researchers. *Journal of public health research* 2016;5(1):jphr. 2016.667.

For peer review only

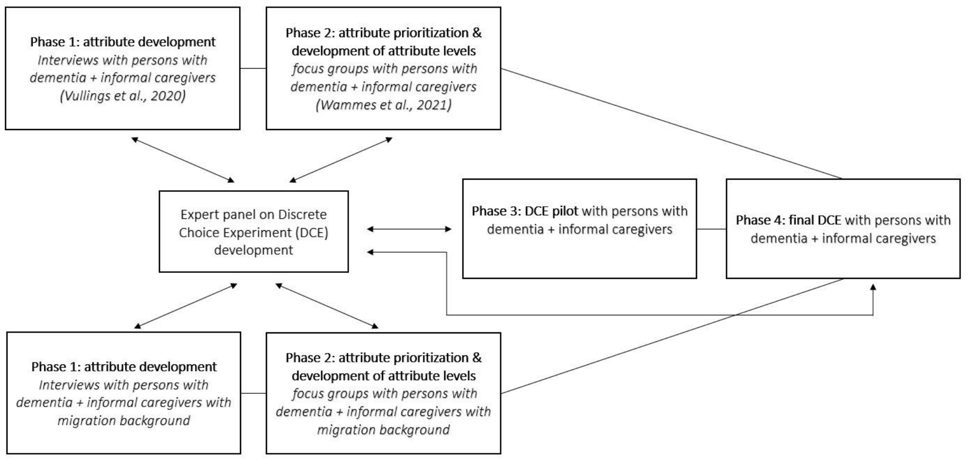

Figure 1: Flowchart study design to create healthcare packages based on discrete choice experiments.

378x184mm (96 x 96 DPI)

	Package A	Package B
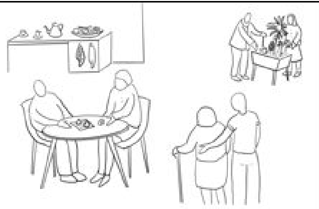 I can participate in activities that I like and socialize with other people	**Once per week** activities are organized **at a daycare center**	**Multiple times per week** there is someone **coming to my house** to do activities together
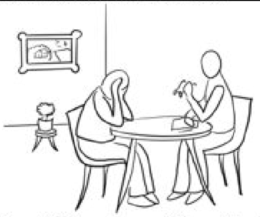 I can talk to someone if I want to share my worries or concerns.	I can talk to a **peer support** group	I can talk to my **case manager**
Select your preference	☐	☐

Figure 2: example of an illustrated DCE choice task

184x114mm (96 x 96 DPI)

BMJ Open

Eliciting preferences of persons with dementia and informal caregivers to support ageing in place in the Netherlands: A protocol for a discrete choice experiment.

Journal:	*BMJ Open*
Manuscript ID	bmjopen-2023-075671.R1
Article Type:	Protocol
Date Submitted by the Author:	02-Nov-2023

Complete List of Authors:	Vullings, Isabelle; Amsterdam UMC Locatie AMC, Geriatrics; Amsterdam Public Health Research Institute<br>Wammes, Joost; Amsterdam UMC Locatie AMC, Geriatrics; Amsterdam Public Health Research Institute<br>Uysal-Bozkir, Özgül; Erasmus University Rotterdam Erasmus School of Social and Behavioural Sciences, Department of Psychology, Education and Child Studies<br>Smits, Carolien; Pharos center of expertise on Health disparities<br>Labrie, Nanon ; Vrije Universiteit Amsterdam, department of language, literature & communication<br>Swait, J.D.; Erasmus University Rotterdam, Erasmus Choice Modelling Centre; Erasmus University Rotterdam, Erasmus School of Health Policy & Management<br>de Bekker-Grob, Esther; Erasmus University Rotterdam, Erasmus Choice Modelling Centre; Erasmus University Rotterdam, Erasmus School of Health Policy & Management<br>Macneil-Vroomen , Janet; Amsterdam Universitair Medische Centra, Geriatrics ; Amsterdam Public Health Research Institute

**Primary Subject Heading**:	Health policy
Secondary Subject Heading:	Geriatric medicine, Health economics, Public health
Keywords:	Dementia, Patient Participation, Decision Making

SCHOLARONE™
Manuscripts

Eliciting preferences of persons with dementia and informal caregivers to support ageing in place in the Netherlands: A protocol for a discrete choice experiment.

Isabelle Vullings[1-2], Joost Wammes[1-2], Özgül Uysal-Bozkir[3], Carolien Smits[4], Nanon Labrie [5], Joffre Swait [6-7], Esther de Bekker-Grob[6-7], Janet L. MacNeil Vroomen [1-2]

1. Department of Internal Medicine, Section of Geriatric Medicine, Amsterdam UMC, Academic Medical Center, University of Amsterdam, Amsterdam, The Netherlands.
2. Amsterdam Public Health research institute, Aging & Later Life, Amsterdam, The Netherlands
3. Erasmus School of Social and Behavioural Sciences, Department of Psychology, Education and Child Studies, Erasmus University Rotterdam, Netherlands.
4. Pharos Center of Expertise on Health Disparities, Utrecht, The Netherlands
5. Department of Language, Literature & Communication, Vrije Universiteit Amsterdam, Amsterdam, The Netherlands
6. Erasmus School of Health Policy & Management, Erasmus University Rotterdam, Rotterdam, The Netherlands.
7. Erasmus Choice Modelling Centre, Erasmus University Rotterdam, Rotterdam, The Netherlands.

Corresponding author:
Msc Isabelle Vullings, i.vullings@amsterdamumc.nl
Department of Internal Medicine, Section of Geriatric Medicine, Amsterdam UMC, Academic Medical Center, University of Amsterdam, Meibergdreef 9, 1105 AZ, Amsterdam, The Netherlands. Amsterdam Public Health research institute, Aging & later life, Amsterdam, The Netherlands.

Keywords: dementia, patient participation, decision making, discrete choice experiment, ageing in place, informal caregivers

Word count: 4201

For peer review only

ABSTRACT

Introduction: Ageing in place (AIP) for persons with dementia is encouraged by European governments and societies. Healthcare packages may need reassessment to account for the preferences of care funders, patients, and informal caregivers. By providing insight into people's preferences, Discrete Choice Experiments (DCEs) can help develop consensus between stakeholders. This protocol paper outlines the development of a Dutch national study to co-create a healthcare package design methodology built on DCEs that is person-centered and helps support informal caregivers and persons with dementia to AIP. A subpopulation analysis of persons with dementia with a migration background is planned due to their high risk for dementia and underrepresentation in research and care.

Methods and analysis: The DCE is designed to understand how persons with dementia and informal caregivers choose between different healthcare packages. Qualitative methods are used to identify and prioritize important care components for persons with dementia to AIP. This will provide a list of care components that will be included in the DCE, to quantify the care needs and preferences of persons with dementia and informal caregivers. The DCE will identify individual and joint preferences to AIP. The relative importance of each attribute will be calculated. The DCE data will be analysed with the use of a random parameters logit model.

Ethics and dissemination: Ethics approval was waived by the Amsterdam University Medical Center (W23_112 #23.137). A study summary will be available on the websites of Alzheimer Nederland, Pharos, and Amsterdam Public Health institute. Results are expected to be presented at [inter]national conferences, peer-reviewed papers will be submitted, and a dissemination meeting will be held to bring stakeholders together. The study results will help improve healthcare package design for all stakeholders.

STRENGTHS AND LIMITATIONS OF THE STUDY

- This will be the first study to include informal caregivers and persons with dementia to identify AIP preferences that has been co-designed with health insurers, policymakers, patient advocacy groups, healthcare professionals, researchers, informal caregivers, and persons with dementia.
- Innovative and rigorous economic methods will be employed to evaluate AIP preferences.
- Pooling of persons with dementia with different migration background may lead to better understanding of their needs as they are typically underrepresented in research.
- A limitation of this study is the generalizability of results as not all migrant groups living in the Netherlands may be included.
- This study is conducted in the Netherlands, findings might be specific to the Dutch Healthcare setting.

For peer review only - http://bmjopen.bmj.com/site/about/guidelines.xhtml

INTRODUCTION

Nearly every country across Europe has reformed its long-term care policy to emphasize ageing in place (AIP) as a way to control costs associated with population ageing (1). The Netherlands is the highest spender of long-term care per gross domestic product in the world (2). Most Dutch long-term care services are paid by the national health insurance, with a low percentage of co-payments (3). To cope with the rising long-term care costs, the Netherlands reformed its long-term care policy in 2015 (4). The reform increased informal care duties and encouraged older adults to AIP (4, 5). A substantial component of the Dutch long-term care costs is attributed to dementia care, as it is the most costly disease encompassing 10.6% of the total Dutch healthcare budget (6). The dementia population is known for its requirement of complex and costly care (7). However, it remains unknown how AIP has affected this population. In the years after the reform, preliminary results indicate an increased need for support with crisis situations such as, unplanned hospitalizations, acute nursing home admissions, and hospital deaths (8, 9). These crises situations are not only costly, but can also compromise the safety and well-being of persons with dementia AIP, in addition to the informal caregivers expected to carry the load.

Despite the need for evidenced-based policy to ensure proper healthcare services, people with dementia and informal caregivers are frequently excluded from policy creation; therefore, services often do not fit with their needs or are not provided at the right time (10, 11). To support successful AIP, identifying the care preferences of persons with dementia and informal caregivers is crucial. Whilst identifying these preferences, special attention should be given to persons with dementia and informal caregivers with a migration background, who are known to have a higher risk of developing dementia (12, 13). Despite their high risk, persons with a migration background are underrepresented in research and care (14). It is crucial that persons with a migration background be included in research so healthcare package evidence is representative of the dementia population. To provide recommendations for healthcare packages it is subsequently needed to quantify the identified care preferences, which can be realized with a discrete choice experiment (DCE).

DCEs are a popular stated preference method used to elicit patients' preferences in healthcare on a large scale (15-17). In a DCE participants are presented with a series of alternative hypothetical scenarios. Participants are repetitively asked to select in each alternative hypothetical scenario their preferred option from amongst a presented set of options, e.g., care packages (16). These choice options are characterized by their attributes and corresponding attribute levels (15, 16). An example of an attribute could be emotional support, described as the possibility to talk to someone about personal feelings or concerns. The corresponding attribute levels could be *(1)* peer support group, *(2)* psychologist or *(3)* telephone helpline. The outcomes (choices) from DCEs are analyzed based on the assumption that participants act in a utility maximizing manner, choosing their most preferred option based on the relative overall attractiveness of the included attributes and attribute levels (16, 18). This protocol paper will outline the development of a Dutch national study to create a healthcare package design methodology, built on DCEs, that is person-centered and helps support both informal caregivers and persons with dementia to successfully AIP. Qualitative methods will be used to identify the care needs and preferences of persons with dementia and their informal caregivers. The results of the qualitative studies will provide insights into the attributes (i.e., care components) that will be included in the DCE. For the DCE, a subpopulation analysis of persons with dementia with a migration background is planned.

For peer review only

Aim

The aim of this study is to identify individual and joint preferences of persons with dementia and informal caregivers for in-home support that enables AIP. We also aim to create optimal healthcare packages for persons with dementia and informal caregivers to AIP. For this second aim, choice model inferences will be the basis for creating the most preferred care packages and testing the uptake in a subsample of participants. This will validate the national model inferences and provide policymakers with high quality, understandable and implementable evidence.

METHODS

Overview of the discrete choice experiment

In this protocol paper, the different phases of designing an inclusive DCE study in which persons with dementia and their informal caregivers can participate will be outlined. The study will be conducted between April 2022 and May 2025. Figure 1 illustrates that the first phase is focused on attribute development, in which semi-structured interviews are used to identify important components of care for persons with dementia and informal caregivers AIP. In the second phase persons with dementia and informal caregivers are asked which of the care components are most important to them, leading to a prioritization of the attributes and the development of attribute levels. For phases one and two, the COREQ guidelines for reporting qualitative research will be followed to ensure important aspects of the research team, reflexivity, study design, findings, and analysis are reported (19). The third phase consists of a pilot which is used to test if the content of the DCE is understandable for persons with dementia and informal caregivers, and if the list of included attributes and corresponding attribute levels is complete. Finally, in phase four the researchers conduct the DCE study. An expert panel will be consulted during all the different phases of the study to reflect upon the research design and findings. Phases three and four will be reported according to the STROBE guidelines, developed as a tool for authors to ensure high-quality reporting of observational studies (20). For constructing the DCE design the ISPOR good research practices reports will also be followed (21, 22).

Patient and public involvement

The DCE, including the framing of the questions, will be developed after focus group discussion and multiple individual interviews with persons with dementia and informal caregivers. Persons with dementia, informal caregivers, and policymakers are involved from the start of the study to ensure the appropriateness of the methodology and to help come to meaningful and implementable results (23). Study materials, such as interview guides, will be discussed with persons with dementia and informal caregivers before the start of the study to ensure appropriateness and make necessary changes. Prior to the DCE, person with dementia, informal caregivers, and patient advocacy groups will be asked to voice their opinion about the final list of attributes and corresponding levels included in the DCE. Patient advocacy groups, persons with dementia and informal caregivers are to be included to make sure that the study properly represents these groups, and the study results reflect their needs and preferences. The patient advocacy groups and policymakers are consulted through an expert panel, details of which can be found in the next section. The GRIPP2 checklist will be followed to report the patient and public involvement (24).

The DCE study will identify individual and joint preferences to AIP, allowing for this concept to be implemented elsewhere. The findings of this study will be published in peer-reviewed journals and an easy-to-understand summary of the results will be available through patient organizations such as Alzheimer Nederland and Pharos. Furthermore, the researchers will organize informative events to communicate the study results to persons with dementia and informal caregivers. These events will

For peer review only - http://bmjopen.bmj.com/site/about/guidelines.xhtml

be organized together with the patient advocacy groups and informal caregivers. Finally, a policy brief will be written to ensure that the study results are known to policy creators and can help to improve healthcare package design for older adults and informal caregivers.

Expert panel

Throughout the entire study, an expert panel will be consulted on the design and content of the DCE, and on the inferences made from its execution. The expert panel will include researchers who have experience with doing DCEs, policymakers, health insurers, patient advocacy groups, persons with dementia and informal caregivers. The experienced researchers will be asked to provide guidance and feedback on the methodological choices made in the DCE design. Subsequently, the envisioned outcome of the DCE study will be discussed with policymakers and health insurers to gain insights concerning the comprehension and appropriateness of this envisioned outcome. The inclusion of policymakers and health insurers in this phase will allow for the opportunity to make changes to the study design that help to realize implementation.

Participants

This study aims to identify preferences of persons with dementia and informal caregivers aging in place, therefore persons with dementia who live in a long-term care facility will not be included in this study. Additional Exclusion criteria will consist of (a) persons who are cognitively impaired to the extent that no conversation can be held with them; (b) people who are unable to provide informed consent. It is know that dementia diagnoses are often delayed, especially in migration populations (25), therefore people without a formal diagnoses will not be excluded a-priori. However, recruiting participants trough healthcare professionals, day-care centers and social organizations that serve persons with dementia and informal caregivers will ensure that all persons have a dementia indication. For the final DCE, the telephone-based interview for cognitive screening (TICS) will be administered to describe the study participants (26).

Prior to the interviews, focus groups, or DCE choice tasks, the researchers will go through the information letter and informed consent form together with the participants to ensure proper understanding and provide the opportunity to ask for clarifications. Subsequently, participants will be asked to provide written informed consent. If participants are unable to write, verbal continued informed consent will be used (27). Throughout the study, participants will be continuously reminded that they are participating in research to ensure that they remain informed and are comfortable to continue (27). It will be stressed that participation can be stopped at any moment.

Attribute and level development for Dutch persons with dementia and informal caregivers

Study phases one and two are already partly completed, as interviews and focus groups were held with persons with dementia and informal caregivers across the Netherlands to identify attributes and levels (Table one) (28, 29). The identified attributes and levels were similar for persons with dementia and informal caregivers, however they prioritized the care components differently (29). People with dementia prioritized day-to-day help and social care, while for informal caregivers, priorities were information about dementia, organisation of care, and emotional support (29). Unfortunately, no persons with a migration background were included in these qualitative studies. Therefore, the interviews and focus groups will be repeated with persons with dementia and informal caregivers with a migration background to ensure the attributes and levels are applicable to this part of society as they have a higher risk for dementia compared to people with non-migration background (12).

For peer review only

Table 1. Attribute description and corresponding levels		
**Attribute**	**Description**	**Possible levels**
In-home care	I can get assistance at home with personal care such as showering, dressing, or medication	- Daily on a fixed time<br>- 24/7 on demand
Help with daily activities	I can get assistance at home with household tasks such as groceries, laundry, cooking, cleaning, or help with doing my finances	- Once per week<br>- Multiple times per week
Social activities	I can participate in social activities that I like to do	- At a day-care facility<br>- At home
Emotional support	I can talk to someone when I feel down or want to share my worries	- Peer support group<br>- Psychologist<br>- Telephone helpline<br>- Case manager
Information about dementia	I can get information about having dementia	- Telephone helpline<br>- Case manager
Navigating the healthcare system	I can get assistance with organizing care, and help with insurances	- Telephone helpline<br>- Case manager
Home adaptations and tools	I can get home adaptations and tools such as a stair lift, grips in shower and toilet, or a personal alarm	- No reimbursements<br>- Fully reimbursed

Note: current list of attributes may need to be updated after expert panel review and interviews with persons with dementia and informal caregivers with a migration background.

Attribute identification for persons with dementia and informal caregivers with a migration background

To identify care preferences and needs of persons with dementia and informal caregivers with a migration background, semi-structured interviews will be performed across the Netherlands. Participants will primarily include persons with dementia and informal caregivers with a Turkish, Moroccan, or Surinamese background as these are the largest groups of non-western immigrants in the Netherlands and they are known to have a high risk for developing dementia (12). People living with dementia who do not speak Dutch as their first language will be welcome to participate.

Participants will be recruited with the help of day-care centers, general practitioners, and organizations that serve persons with a migration background (Pharos and NOOM). The organizations will inform potential participants about the study, and interested participants will be provided with a bilingual invitation letter. The interviews will be conducted in the preferred language of the participants: Turkish, Moroccan-Arabic, Tarifit, or Dutch (12). Interviews will be conducted by trained bilingual interviewers. Persons with dementia and informal caregivers will be interviewed separately. However, since it is important that participants feel comfortable, if they strongly wish to be interviewed together this will be permitted.

The interviews will be semi-structured, which means that an interview guide (supplementary file 1) will be used to provide some structure, whilst leaving room for new topics to be brought up by the participants. The interview guide will generally be based on the guide (supplementary file 2) of the interview study for persons with non-migration backgrounds (28), with additions based on findings of previous studies that focused on persons with dementia and informal caregivers with a migration background (30, 31). The cultural sensitivity and understandability of the interview guide will be assessed by Pharos, center of expertise in health disparities, and experienced researchers (CS and ÖUB). The interviews will be transcribed and translated by the bilingual interviewer who conducted

For peer review only

the interviews. Respondents will have the opportunity to review the translated transcripts, which will help to ensure proper interpretation and provide opportunities to give clarifications. It will be stressed that this is voluntary, participants are free to choose if they wish to read the translated transcripts. Interviews will be held until data saturation is reached, which is expected after approximately 10 interviews for each group (32). Thematic analysis will be used to analyze and identify relevant patterns within the data (33). Coding of the data will be done with both a deductive and inductive approach, starting with certain theoretical or empirical assumptions, with an open approach (33). The results will provide a list of attributes (care components) that are important for persons with dementia and informal caregivers with a migration background.

Attribute prioritization and the development of attribute levels

To understand the relative importance of the care components that are identified, mixed focus group sessions will be held with a new sample of persons with dementia and informal caregivers with a Turkish, Moroccan, or Surinamese background (12). Focus groups create an environment in which participants can share their experiences and build on each other's knowledge (29). To ensure a free discussion, informal caregivers will not be participating in the same focus group as their person with dementia (29). Participants will be recruited with the help of previously mentioned organizations. To facilitate dialogue between the participants, focus groups will include participants with similar migration backgrounds and linguistic preferences. The focus groups will be conducted by trained bicultural interviewers. The focus group session will include a quantitative ranking exercise in which persons with dementia and informal caregivers will be asked to list the care components from most important to least important (29). For this ranking exercise, cards with the previously identified care characteristics, containing visual and written information will be used. During the focus group, the reasoning behind the choices made in the ranking exercise will be discussed. Focus groups will be held until saturation is reached, which is expected after approximately six sessions, each including persons with dementia (n = 4) and informal caregivers (n = 4). Focus groups will be transcribed and translated by the trained bilingual interviewers. Participants will be provided with the opportunity to read the translated transcripts and, if necessary, provide clarifications. Thematic analysis will be used to analyze and identify relevant patterns within the data (33). Coding of the data will be done with both a deductive and inductive approach, starting with certain theoretical or empirical assumptions, with an open approach (33). The results will consist of a ranking of the attributes (care components) and a description of the attribute levels (characteristics of care components).

Discrete choice experiment pilot

For the DCE pilot the list of attributes that has previously been identified (Table 1) will be updated with the findings of the qualitative studies with persons with dementia and informal caregivers with a migration background. The pilot will include both individual DCE rounds with the persons with dementia (n=4) and informal caregivers (n=4), and dyadic rounds with both the person with dementia and their informal caregiver. The in-person pilot will include the testing of respondents' understanding of choice tasks and the appropriateness of the included attributes and attribute levels. Furthermore, the pilot will help to illustrate if the length of the DCE survey is acceptable. Based on the pilot, changes might need to be made to the DCE survey to increase appropriateness and understandability, as well as manage survey complexity for persons with dementia.

For peer review only

The final discrete choice experiment

The final DCE will include three rounds: (1) persons with dementia, (2) informal caregivers, and (3) dyadic rounds with both the persons with dementia and informal caregivers. The first two rounds will help to elucidate individual preferences, whilst the dyadic round will help to elucidate joint preferences. Identifying joint preferences is important since decisions about care are often made by multiple individuals (34-36). Furthermore, the dyadic DCE round can pose as an opportunity for the person with dementia and their informal caregiver to have an in-depth conversation about their preferences and needs, which could lead to better mutual understanding (37). It is likely that preference adaptation will occur, however as shown in previous research this adaptation can be from both the person with dementia and the informal caregiver (37). Informal caregivers were not primarily dominant in dyadic DCE rounds, in fact the informal caregivers were found to be helpful in assisting the person with dementia to complete more choice tasks (37). An active role of the researcher administering the DCE choice tasks is needed to help guide the dialogue in the joint DCE rounds. People with dementia will be encouraged to state their preference and motivation first to minimize agency of informal caregivers (37).

The complexity of a DCE can be challenging for persons with dementia (38-40). However, a recent study found that persons with moderate cognitive impairment can complete DCE choice tasks (37). Therefore, persons will not be excluded a-priori based on their level of cognitive impairment. The following options in the DCE design will help to lower the cognitive burden for persons with dementia. First, illustrations will be used to visualize the choice sets and included attributes. Figure 2 provides an example of such an illustration (37). The illustrations are made by a graphic designer who has experience illustrating pictures for persons with cognitive impairments. Second, the DCE will have binary choice tasks, asking participants to choose between packages A and B, instead of including multiple alternatives (e.g., additional packages C or D). Participant will complete a maximum of six choice tasks, consisting of three attributes and two corresponding levels (37). Blocked fractional factorial designs (41) will be created with the use of Ngene. Finally, the DCE will start with a practice round to test decision making skills (37). Persons with dementia will be asked to think aloud whilst completing the choice tasks. The think-aloud technique is commonly used in research to make thought processes of participants observable for researchers (42). It has been found useful in supporting persons with dementia to complete DCE choice tasks, as it enables researchers to observe difficulties and, if needed, provide guidance or remind persons with dementia about the rules of the choice tasks (37).

Power calculation

Envisioning a two-alternative choice task and four replicates per dyad, a minimum sample size $N \geq 576$ is recommended for a discrete choice experiment to reliably achieve 95% confidence intervals on true uptake probabilities of 0.4 (43). Therefore, the goal will be to achieve a sample size of 600 dyads of persons with dementia and informal caregivers, covering all targeted groups. Purposive sampling of minority groups, people living in urban and rural areas, and people with different educational levels will be used. In addition, sampling across the country will help to make the healthcare package as nationally representative as possible (44).

Participants will be recruited through general practitioners, geriatricians, day-care facilities, and organizations that serve persons with dementia and informal caregivers (Alzheimer Nederland, Pharos, NOOM). The professionals will inform potential participants about the study, and provide them with the (bilingual) information letter. With the participants' consent, the researchers will obtain their

For peer review only

contact information form the professionals. The researchers will contact the potential participant by telephone to answer any remaining questions and to administer the TICS (26). The researchers will schedule an appointment at the participants home to complete the DCE choice tasks in person.

Outcomes

The main outcome of the DCE study will be the relative importance of the attributes (care components). The relative importance of each attribute will be calculated as the difference between the preference weights of the most and least preferred level of that attribute (45). The relative importance will be scaled so that 10 indicates the most preferred attribute. The difference between individual and joint DCE round will be analysed with summary statistics and bivariate comparisons.

A contingent valuation approach will be used to identify the participants' willingness to pay (WTP) for the most preferred programs outside of the DCE (46, 47). While out-of-pockets costs for social care does not currently occur in the Netherlands yet, this may soon change. Therefore, an indication of the WTP is helpful for policy makers and health insurers. At the end of the DCE, participants indicate their preferred envisioned package over their current service provision, they will be asked if they are willing to pay an additional monthly out-of-pocket fee of €50 for this package. If participants answer this question with yes, additional questions with out-of-pocket fees of €100 and €200 will be asked (46). If participants answered no to the initial question of €50, similar questions with an amount of €25 and €12,50 will be asked (46). Finally, participants will be asked to indicate their maximum WTP in an open-ended question (46). For the analysis, the maximum price expressed in the open-ended question will be used and summarized through the median and interquartile range (46).

Statistical analysis

The DCE data will be analysed with a random parameters logit (RPL) model (45). For each attribute level, coefficient estimates will be estimated from the RPL model, which can be interpreted as the relative preference weight (45). Individual and joint models will be created to evaluate preferences of persons with dementia and informal caregivers at the individual and joint DCE rounds.

To analyse if persons with dementia and informal caregivers with a migration background have different preferences, post hoc subgroup analysis will be conducted. Dummy coded variables will be added to the RPL model to identify participants that are part of given subgroups. The attribute levels in the RPL model will be interacted with the dummy variable, and all interaction terms will be added to the original RPL model (45). The estimated parameters of the interaction terms will illustrate the difference in preferences between the subgroup (persons with a migration background) and the reference group (persons without a migration background)(45). This method can also be used to analyse if there are different preferences based on other subgroups, such as socio-economic status.

Finally, the attributes with the highest relative importance can be used to build most preferred programs (healthcare packages). To ensure validity of these results for health insurers and government, the uptake of the most preferred programs will be tested on a subsample of the target population. Model predictions will be verified by creating the most preferred programs according to the preferences of persons with dementia and informal caregivers that were found in the DCE. A new sample will be collected of 50-60 participants to test the uptake of the most preferred programs by asking participants which one they would rather have over their current service provision (48).

DISCUSSION

The discrete choice methodology described in this paper enables persons with dementia and informal caregivers to participate in policy formulation and evaluation. It is assumed that this will help to avoid

crisis situations and improve the quality of life of persons with dementia and their informal caregivers. Furthermore, the outcome of the final DCE will be a ranking of the most preferred care components represented in a care package. This helps health insurers and the ministry of health to maximize their chances for designing healthcare packages that are appropriate, useful, and meaningful.

Furthermore, this study protocol provides an important methodological contribution. It is a clear guide on how to build an inclusive DCE, covering the important methodological choices throughout the required study phases. Moreover, it provides guidelines on how to include underrepresented groups in DCEs. The methodological choices described to help include persons with dementia with a migration background, can be used to include other underrepresented groups such as people with low (health) literacy (49, 50). Finally, this study provides an example of how to overcome language barriers in research and knowledge dissemination.

Strengths and limitations of this study

A strength of this study will be that it is the first study to include informal caregivers and persons with dementia to identify AIP preferences that has been co-designed with health insurers, policymakers, patient advocacy groups, healthcare professionals, researchers, informal caregivers, and persons with dementia. Innovative and rigorous economic methods will be employed to evaluate AIP preferences. Another strength is the inclusion of persons with dementia with different migration backgrounds, which can lead to a better understanding of their needs since they are typically underrepresented in research.

A limitation of this study is the generalizability of results, as not all migrant groups living in the Netherlands may be included. In addition, the chosen recruitment strategies make it unlikely for persons who do not have a diagnoses to be included in this study. Persons with communication difficulties, such as hearing or vision loss will have more difficulties to participate in DCEs. However, the participants ability to complete the DCE will be assessed with practice choice tasks. Finally, enabling persons to participate from their own homes helps to include persons with limited mobility. The research team strives to make the study as inclusive as possible.

Ethics and dissemination

Based on the study protocol, the Ethics Committee (METC) of the Amsterdam University Medical Centre waived the obligation for the study to undergo formal ethical approval as is described under Dutch law in the Medical Research in Humans Act, January 2019 (ref W23_112 #23.137).

Consent

This is a prospective study and pseudonymised data is used, written and continued informed consent will be obtained from the participants prior to participation. This is consistent with current European legislation under the General Data Protection Regulation (GDPR). This study will abide by the Declaration of Helsinki and present ethical requirements.

Authors' contributions

JMV conceptualized the study and developed the study design together with the other researchers. IV and JW contributed to the development of the study design, data collection and analysis. The authors provided input for the study design from their own area of expertise; inclusive research (CS & ÖUB), qualitative research (NL), discrete choice experiments (EBG & JS). IV wrote the manuscript, the co-authors read, edited, and approved the final version.

For peer review only

Funding statement
This work was supported by Alzheimer Nederland, (WE,06-2021-04). In addition, grant support was from The Netherlands Organisation for Scientific Research (https://www.nwo.nl/en) (NWO-Talent-Scheme-Vidi-Grant No. 09150171910002) to EBG.

Competing interest statement
No competing interests to report.

Figure legends
Figure 1. Flowchart study design
Caption: Figure 1: Flowchart study design to create healthcare packages based on discrete choice experiments
Figure 2: Illustrated DCE choice tasks
Caption: Figure 2: Example of an illustrated DCE choice task

For peer review only

References

1. Spasova S, Baeten, R., Coster, S., Ghailani, D., Peña-Casas, R. and Vanhercke, B. Challenges in long-term care in Europe. A study of national policies. Brussels: European Commission, (ESPN) ESPN; 2018.
2. Organisation for Economic Cooperation and Development. long-term care spendings and unit costs Paris: OECD publishing 2021.
3. Bakx P, Schut E, Wouterse B. Price setting in long-term care in the Netherlands. Erasmus School of Health Policy and Management, November. 2020.
4. Alders P, Schut FT. The 2015 long-term care reform in the Netherlands: Getting the financial incentives right? Health Policy. 2019;123(3):312-6.
5. Maarse JH, Jeurissen PP. The policy and politics of the 2015 long-term care reform in the Netherlands. Health Policy. 2016;120(3):241-5.
6. RIVM. Effecten van vergrijzing steeds beter zichtbaar in uitgaven gezondheidszorg 2022 [Available from: https://www.rivm.nl/nieuws/effecten-van-vergrijzing-steeds-beter-zichtbaar-in-uitgaven-gezondheidszorg#:~:text=In%202019%20werd%20in%20Nederland,naar%20de%20kosten%20van%20ziekten.
7. Wimo A, Guerchet M, Ali G-C, Wu Y-T, Prina AM, Winblad B, et al. The worldwide costs of dementia 2015 and comparisons with 2010. Alzheimer's & Dementia. 2017;13(1):1-7.
8. Francke A, Heide I, Bruin Sd, Gijsen R, Poos R, Verbeek M, et al. Een samenhangend beeld van dementie en dementiezorg: kerncijfers, behoeften, aanbod en impact. Themarapportage van de Staat van Volksgezondheid en Zorg. 2018.
9. MacNeil-Vroomen JL, van der Steen JT, Holman R, Monin JK, Buurman BM. Hospital deaths increased after reforms regardless of dementia status: an interrupted time-series analysis. Journal of the American Medical Directors Association. 2021;22(7):1507-11.
10. Van der Roest HG, Meiland FJ, Comijs HC, Derksen E, Jansen AP, Van Hout HP, et al. What do community-dwelling people with dementia need? A survey of those who are known to care and welfare services. International psychogeriatrics. 2009;21(5):949-65.
11. Van Der Roest HG, Meiland FJ, Maroccini R, Comijs HC, Jonker C, Dröes R-M. Subjective needs of people with dementia: a review of the literature. International Psychogeriatrics. 2007;19(3):559-92.
12. Parlevliet JL, Uysal-Bozkir Ö, Goudsmit M, van Campen JP, Kok RM, Ter Riet G, et al. Prevalence of mild cognitive impairment and dementia in older non-western immigrants in the Netherlands: a cross-sectional study. Int J Geriatr Psychiatry. 2016;31(9):1040-9.

13. Selten J-P, Termorshuizen F, van Sonsbeek M, Bogers J, Schmand B. Migration and dementia: a meta-analysis of epidemiological studies in Europe. Psychological Medicine. 2021;51(11):1838-45.
14. Schmachtenberg T, Monsees J, Hoffmann W, van den Berg N, Stentzel U, Thyrian JR. Comparing national dementia plans and strategies in Europe–is there a focus of care for people with dementia from a migration background? BMC Public Health. 2020;20:1-10.
15. de Bekker-Grob EW, Ryan M, Gerard K. Discrete choice experiments in health economics: a review of the literature. Health economics. 2012;21(2):145-72.
16. Soekhai V, de Bekker-Grob EW, Ellis AR, Vass CM. Discrete choice experiments in health economics: past, present and future. Pharmacoeconomics. 2019;37(2):201-26.
17. Friedel JE, Foreman AM, Wirth O. An introduction to "discrete choice experiments" for behavior analysts. Behavioural processes. 2022;198:104628.
18. Clark MD, Determann D, Petrou S, Moro D, de Bekker-Grob EW. Discrete choice experiments in health economics: a review of the literature. Pharmacoeconomics. 2014;32(9):883-902.
19. Tong A, Sainsbury P, Craig J. Consolidated criteria for reporting qualitative research (COREQ): a 32-item checklist for interviews and focus groups. International journal for quality in health care. 2007;19(6):349-57.
20. Cuschieri S. The STROBE guidelines. Saudi J Anaesth. 2019;13(Suppl 1):S31-s4.
21. Johnson FR, Lancsar E, Marshall D, Kilambi V, Mühlbacher A, Regier DA, et al. Constructing experimental designs for discrete-choice experiments: report of the ISPOR conjoint analysis experimental design good research practices task force. Value in health. 2013;16(1):3-13.
22. Bridges JF, de Bekker-Grob EW, Hauber B, Heidenreich S, Janssen E, Bast A, et al. A roadmap for increasing the usefulness and impact of patient-preference studies in decision making in health: A good practices report of an ISPOR task force. Value in Health. 2023;26(2):153-62.
23. Illes J, Lawson A, McDonald PJ. Ethical Considerations for Discrete Choice Experiments with Caregivers. Journal of Empirical Research on Human Research Ethics. 2022;17(4):426-30.
24. Staniszewska S, Brett J, Simera I, Seers K, Mockford C, Goodlad S, et al. GRIPP2 reporting checklists: tools to improve reporting of patient and public involvement in research. BMJ. 2017;358:j3453.
25. Goudsmit M, Uysal-Bozkir Ö, Parlevliet JL, van Campen JP, de Rooij SE, Schmand B. The Cross-Cultural Dementia Screening (CCD): A new neuropsychological screening instrument for dementia in elderly immigrants. Journal of Clinical and Experimental Neuropsychology. 2017;39(2):163-72.
26. Brandt J, Spencer M, Folstein M. The telephone interview for cognitive status. Neuropsychiatry Neuropsychol Behav Neurol. 1988;1(2):111-7.
27. McKeown J, Clarke A, Ingleton C, Repper J. Actively involving people with dementia in qualitative research. Journal of clinical nursing. 2010;19(13-14):1935-43.
28. Vullings I, Labrie N, Wammes JD, de Bekker-Grob EW, MacNeil-Vroomen J. Important components for Dutch in-home care based on qualitative interviews with persons with dementia and informal caregivers. Health Expectations. 2020;23(6):1412-9.
29. Wammes JD, Labrie NH, Agogo GO, Monin JK, de Bekker-Grob EW, MacNeil Vroomen JL. Persons with dementia and informal caregivers prioritizing care: A mixed-methods study. Alzheimer's & Dementia: Translational Research & Clinical Interventions. 2021;7(1):e12193.
30. Lillekroken D, Halvorsrud L, Gulestø R, Bjørge H. Family caregivers' experiences of providing care for family members from minority ethnic groups living with dementia: A qualitative systematic review. Journal of Clinical Nursing. 2021.
31. Duran-Kiraç G, Uysal-Bozkir Ö, Uittenbroek R, van Hout H, Broese van Groenou MI. Accessibility of health care experienced by persons with dementia from ethnic minority groups and formal and informal caregivers: A scoping review of European literature. Dementia. 2022;21(2):677-700.
32. Hennink MM, Kaiser BN, Marconi VC. Code saturation versus meaning saturation: how many interviews are enough? Qualitative health research. 2017;27(4):591-608.
33. Braun V, Clarke V. Using thematic analysis in psychology. Qualitative research in psychology. 2006;3(2):77-101.

For peer review only

34. Corfman KP, Lehmann DR. Models of cooperative group decision-making and relative influence: An experimental investigation of family purchase decisions. Journal of Consumer Research. 1987;14(1):1-13.
35. Hensher DA, Beck MJ, Rose JM. Accounting for preference and scale heterogeneity in establishing whether it matters who is interviewed to reveal household automobile purchase preferences. Environmental and Resource Economics. 2011;49(1):1-22.
36. Groen-van de Ven L, Smits C, Span M, Jukema J, Coppoolse K, de Lange J, et al. The challenges of shared decision making in dementia care networks. International Psychogeriatrics. 2018;30(6):843-57.
37. Wammes JD, Swait JD, de Bekker-Grob EW, Monin JK, Labrie NH, MacNeil Vroomen JL. Dyadic Discrete Choice Experiments Enable Persons with Dementia and Informal Caregivers to Participate in Health Care Decision Making: A Mixed Methods Study. Journal of Alzheimer's Disease. 2022(Preprint):1-10.
38. Chester H, Clarkson P, Davies L, Sutcliffe C, Davies S, Feast A, et al. People with dementia and carer preferences for home support services in early-stage dementia. Aging & Mental Health. 2018;22(2):270-9.
39. O'Philbin L, Woods B, Holmes E. People with dementia and caregiver preferences for digital life story work service interventions. A discrete choice experiment and digital survey. Aging & Mental Health. 2020;24(2):353-61.
40. Ratcliffe J, Hutchinson C, Milte R, Nguyen KH, Welch A, Caporale T, et al. How do people with dementia and family carers value dementia-specific quality of life states? An explorative "Think Aloud" study. Australasian Journal on Ageing. 2019;38:75-82.
41. Jaynes J, Wong WK, Xu H. Using blocked fractional factorial designs to construct discrete choice experiments for healthcare studies. Statistics in medicine. 2016;35(15):2543-60.
42. Uysal-Bozkir Ö, Fokkema T, MacNeil-Vroomen JL, van Tilburg TG, de Rooij SE. Translation and validation of the De Jong Gierveld loneliness scale among older migrants living in the Netherlands. The Journals of Gerontology: Series B. 2017;72(1):109-19.
43. Louviere JJ, Hensher DA, Swait JD. Stated choice methods: analysis and applications: Cambridge university press; 2000.
44. Valliant R, Dever JA, Kreuter F. Practical tools for designing and weighting survey samples: Springer; 2013.
45. Rosenow F, Winter Y, Leunikava I, Brunnert M, Joeres L, Sutphin J, et al. Relative importance of clinical outcomes and safety risks of antiseizure medication monotherapy for patients and physicians: Discrete choice experiment eliciting preferences in real-world study" VOTE". Epilepsia. 2022;63(2):451-62.
46. Vo NX, Nguyen TTH, Van Nguyen P, Tran QV, Vo TQ. Using contingent valuation method to estimate adults' willingness to pay for a future coronavirus 2019 vaccination. Value in Health Regional Issues. 2021;24:240-6.
47. Basu R. Willingness-to-pay to prevent Alzheimer's disease: a contingent valuation approach. International journal of health care finance and economics. 2013;13(3):233-45.
48. Janssen EM, Marshall DA, Hauber AB, Bridges JF. Improving the quality of discrete-choice experiments in health: how can we assess validity and reliability? Expert review of pharmacoeconomics & outcomes research. 2017;17(6):531-42.
49. Hughson J-a, Woodward-Kron R, Parker A, Hajek J, Bresin A, Knoch U, et al. A review of approaches to improve participation of culturally and linguistically diverse populations in clinical trials. Trials. 2016;17(1):1-10.
50. Woodward-Kron R, Hughson J-a, Parker A, Bresin A, Hajek J, Knoch U, et al. Culturally and linguistically diverse populations in medical research: perceptions and experiences of older Italians, their families, ethics administrators and researchers. Journal of public health research. 2016;5(1):jphr. 2016.667.

For peer review only

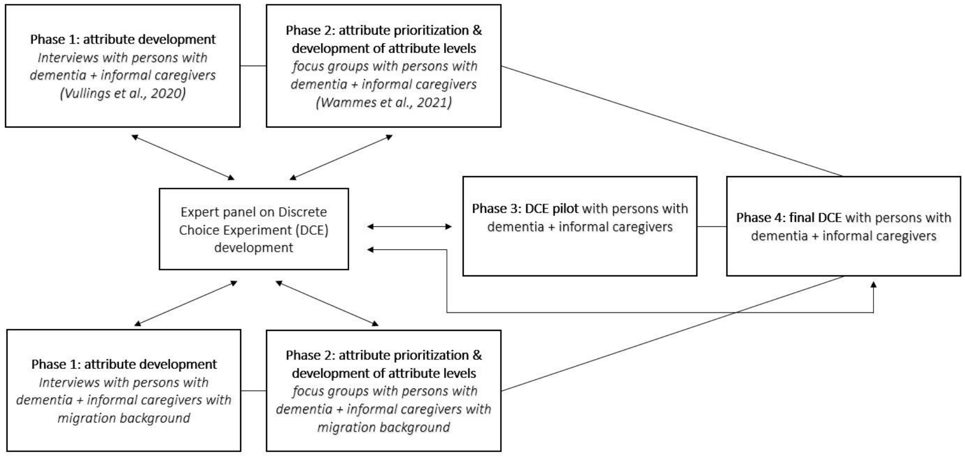

Figure 1: Flowchart study design to create healthcare packages based on discrete choice experiments.

378x184mm (96 x 96 DPI)

	Package A	Package B
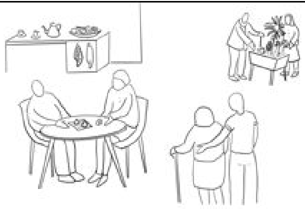 I can participate in activities that I like and socialize with other people	**Once per week** activities are organized **at a daycare center**	**Multiple times per week** there is someone **coming to my house** to do activities together
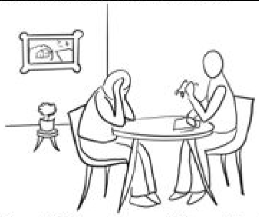 I can talk to someone if I want to share my worries or concerns.	I can talk to a **peer support** group	I can talk to my **case manager**
Select your preference	☐	☐

Figure 2: example of an illustrated DCE choice task

184x114mm (96 x 96 DPI)

Introduction: Thank you for participating in this study. My name is [short introduction interviewer]. The research that we are doing is focused on persons who are living at home. I would like to talk with you about your living situation at home, the daily things that you do and the help that you receive. If I ask a question that is not clear, feel free to let me know. There are no wrong answers, you can say anything that comes to mind. I would like to record the interview, if that is ok with you. The recordings will only be shared with my colleagues. The conversation will not be shared with your doctor or family. We will leave out your name. your name will be replaced by a number. *[reading information letter + informed consent – singing informed consent – start recording]*

Before we start, I have a few general questions that I would like to ask you: 1) What education did you receive? 2) Where were you born? 3) Where were your parents born? 4) How long have you lived in the Netherlands? 5) How old are you? 6) Where do you live? 7) Do you live alone? 8) Where does your family live?

Theme	Explanation theme	Examples of questions
Introduction	Start-up questions giving respondent room to talk and bring up topics.	• Can you tell something about yourself?<br>• How is it going at home?<br>• How is your health?
Daily activities	Talk about: (help with) cleaning, cooking, showering, getting dressed etc.<br><br>Have attention for religious activities praying, going to mosque/church.<br><br>Find out if they get help with these activities, from who, and how they value this help.	• What are daily activities that you do?<br><br>• What does your day look like?
Social activities	Talk about: social activities such as, female/male groups, visiting other persons etc.<br><br>Ask if people go to a daycare facility.<br><br>For informal caregivers: ask if they have time to do things for themselves.	• What are things that you like to do?<br><br>• What are things that you like to do with other people?
Adaptations in the home	Talk about: adaptations such as a stair lift, grips in the shower, alarm system etc.	• What changes are made in your house?<br>• What are things that you would like to change about your house?

For peer review only

Information	Talk about: information received after diagnosis about what to expect or how to cope with dementia.	• Have you gotten information about what to expect (after diagnosis)?<br><br>• What questions do you have about dementia?
Contact with care professionals	Talk about: how people experience contact with care professionals such as home care, general practitioner, and the case manager dementia.	• What do you think of the home care providers?<br><br>• How is your contact with the general practitioner?
Navigating the healthcare system	Talk about: does the person arrange care themselves or do they get help (from family, general practitioner, or case manager).<br><br>Do they struggle with this or not.	• How is the care arranged?<br>• Who arranges this support/care?<br><br>• How would you like to be supported with the arrangement of care?
Emotional support	Talk about: how people feel about their situation, their worries or concerns.<br><br>Do they talk about this and with who.	• How do you feel about your situation (dementia diagnosis)?<br><br>• do you have someone to discuss your concerns with?
Closing questions	Question to end the interview, ask if participants want to add something.	• What are things that you would like to get help with?<br><br>• Are there things that you would like to add?

Examples of probing questions

- With who do you do this? / Who helps you with this?
- How often do you do this? / How often do you get this help?
- What do you like about this help/support?
- What do you dislike about this (help/support)?

Thank you so much for sharing your story. *[ask if people wat to be informed about the results of the study].*

For peer review only

Introduction: Thank you for participating in this study. My name is [short introduction interviewer]. The research that we are doing is focused on persons who are living at home. I would like to talk with you about your living situation at home, the daily things that you do and the help that you receive. If I ask a question that is not clear, feel free to let me know. There are no wrong answers, you can say anything that comes to mind. I would like to record the interview, if that is ok with you. The recordings will only be shared with my colleagues. The conversation will not be shared with your doctor or family. We will leave out your name. your name will be replaced by a number. *[reading information letter + informed consent – singing informed consent – start recording]*

Before we start, I have a few general questions that I would like to ask you: 1) What education did you receive? 2) Where were you born? 3) How old are you? 4) Where do you live? 5) Do you live alone? 6) Where does your family live?

Theme	Explanation theme	Examples of questions
Introduction	Start-up questions giving respondent room to talk and bring up topics.	• Can you tell something about yourself?<br>• How is it going at home?<br>• How is your health?
Daily activities	Talk about: (help with) cleaning, cooking, showering, getting dressed etc.<br><br>Find out if they get help with these activities, from who and how they value this help.	• What are daily activities that you do?<br>• What does your day look like?<br><br>• Do you receive help with these daily activities?<br>• How would you like to be helped with these activities?
Social activities	Talk about: social activities such as visiting other persons, playing cards etc.<br><br>Ask if people go to a daycare facility + how they value this.<br><br>For informal caregivers: ask if they have time for themselves, to do their own things.	• What are things that you like to do?<br>• What are things that you like to do with other people?<br><br>• With who do you like to do these activities?<br>• How would you like to be supported in this?
Adaptations in the home	Talk about: adaptations such as a stair lift, grips in the shower, alarm system etc.	• What changes are made in your house?<br>• What are things that you would like to change about your house?

For peer review only

Information	Talk about: information received after diagnosis.<br><br>Did they get information about what to expect or how to cope with dementia.	• Have you gotten information about what to expect (after diagnosis)?<br>• How was the support of your general practitioner/ case manager dementia?<br><br>• What questions do you have about dementia?
Cognitive training	Talk about: if people use tools to help them remember.	• How do your memory problems affect you?<br><br>• What tools do you use to help you remember?
Emotional support	Talk about: how people feel about their situation, their worries or concerns.<br><br>Do they talk about this and with who.	• How do you feel about your situation (dementia diagnosis)?<br><br>• do you have someone to discuss your concerns with?
Closing questions	Question to end the interview, ask if participants want to add something.	• What are things that you would like to get help with?<br><br>• Are there things that you would like to add?

Examples of probing questions

- With who do you do this? / Who helps you with this?
- How often do you do this? / How often do you get this help?
- What do you like about this help/support?
- What do you dislike about this (help/support)?

Thank you so much for sharing your story. *[ask if people wat to be informed about the results of the study].*

For peer review only

BMJ Open

Eliciting preferences of persons with dementia and informal caregivers to support ageing in place in the Netherlands: A protocol for a discrete choice experiment.

Journal:	*BMJ Open*
Manuscript ID	bmjopen-2023-075671.R2
Article Type:	Protocol
Date Submitted by the Author:	28-Nov-2023
Complete List of Authors:	Vullings, Isabelle; Amsterdam UMC Locatie AMC, Geriatrics; Amsterdam Public Health Research Institute<br>Wammes, Joost; Amsterdam UMC Locatie AMC, Geriatrics; Amsterdam Public Health Research Institute<br>Uysal-Bozkir, Özgül; Erasmus University Rotterdam Erasmus School of Social and Behavioural Sciences, Department of Psychology, Education and Child Studies<br>Smits, Carolien; Pharos center of expertise on Health disparities<br>Labrie, Nanon ; Vrije Universiteit Amsterdam, department of language, literature & communication<br>Swait, J.D.; Erasmus University Rotterdam, Erasmus Choice Modelling Centre; Erasmus University Rotterdam, Erasmus School of Health Policy & Management<br>de Bekker-Grob, Esther; Erasmus University Rotterdam, Erasmus Choice Modelling Centre; Erasmus University Rotterdam, Erasmus School of Health Policy & Management<br>Macneil-Vroomen , Janet; Amsterdam Universitair Medische Centra, Geriatrics ; Amsterdam Public Health Research Institute
**Primary Subject Heading**:	Health policy
Secondary Subject Heading:	Geriatric medicine, Health economics, Public health
Keywords:	Dementia, Patient Participation, Decision Making

SCHOLARONE™
Manuscripts

Eliciting preferences of persons with dementia and informal caregivers to support ageing in place in the Netherlands: A protocol for a discrete choice experiment.

Isabelle Vullings[1-2], Joost Wammes[1-2], Özgül Uysal-Bozkir[3], Carolien Smits[4], Nanon Labrie [5], Joffre Swait [6-7], Esther de Bekker-Grob[6-7], Janet L. MacNeil Vroomen [1-2]

1. Department of Internal Medicine, Section of Geriatric Medicine, Amsterdam UMC, Academic Medical Center, University of Amsterdam, Amsterdam, The Netherlands.
2. Amsterdam Public Health research institute, Aging & Later Life, Amsterdam, The Netherlands
3. Erasmus School of Social and Behavioural Sciences, Department of Psychology, Education and Child Studies, Erasmus University Rotterdam, Netherlands.
4. Pharos Center of Expertise on Health Disparities, Utrecht, The Netherlands
5. Department of Language, Literature & Communication, Vrije Universiteit Amsterdam, Amsterdam, The Netherlands
6. Erasmus School of Health Policy & Management, Erasmus University Rotterdam, Rotterdam, The Netherlands.
7. Erasmus Choice Modelling Centre, Erasmus University Rotterdam, Rotterdam, The Netherlands.

Corresponding author:
Msc Isabelle Vullings, i.vullings@amsterdamumc.nl
Department of Internal Medicine, Section of Geriatric Medicine, Amsterdam UMC, Academic Medical Center, University of Amsterdam, Meibergdreef 9, 1105 AZ, Amsterdam, The Netherlands. Amsterdam Public Health research institute, Aging & later life, Amsterdam, The Netherlands.

Keywords: dementia, patient participation, decision making, discrete choice experiment, ageing in place, informal caregivers

Word count: 4203

ABSTRACT

Introduction: Ageing in place (AIP) for persons with dementia is encouraged by European governments and societies. Healthcare packages may need reassessment to account for the preferences of care funders, patients, and informal caregivers. By providing insight into people's preferences, Discrete Choice Experiments (DCEs) can help develop consensus between stakeholders. This protocol paper outlines the development of a Dutch national study to co-create a healthcare package design methodology built on DCEs that is person-centered and helps support informal caregivers and persons with dementia to AIP. A subpopulation analysis of persons with dementia with a migration background is planned due to their high risk for dementia and underrepresentation in research and care.

Methods and analysis: The DCE is designed to understand how persons with dementia and informal caregivers choose between different healthcare packages. Qualitative methods are used to identify and prioritize important care components for persons with dementia to AIP. This will provide a list of care components that will be included in the DCE, to quantify the care needs and preferences of persons with dementia and informal caregivers. The DCE will identify individual and joint preferences to AIP. The relative importance of each attribute will be calculated. The DCE data will be analysed with the use of a random parameters logit model.

Ethics and dissemination: Ethics approval was waived by the Amsterdam University Medical Center (W23_112 #23.137). A study summary will be available on the websites of Alzheimer Nederland, Pharos, and Amsterdam Public Health institute. Results are expected to be presented at [inter]national conferences, peer-reviewed papers will be submitted, and a dissemination meeting will be held to bring stakeholders together. The study results will help improve healthcare package design for all stakeholders.

STRENGTHS AND LIMITATIONS OF THE STUDY

- This will be the first study to include informal caregivers and persons with dementia to identify AIP preferences that has been co-designed with health insurers, policymakers, patient advocacy groups, healthcare professionals, researchers, informal caregivers, and persons with dementia.
- Innovative and rigorous economic methods will be employed to evaluate AIP preferences.
- Pooling of persons with dementia with different migration background may lead to better understanding of their needs as they are typically underrepresented in research.
- A limitation of this study is the generalizability of results as not all migrant groups living in the Netherlands may be included.
- This study is conducted in the Netherlands, findings might be specific to the Dutch Healthcare setting.

For peer review only - http://bmjopen.bmj.com/site/about/guidelines.xhtml

INTRODUCTION

Nearly every country across Europe has reformed its long-term care policy to emphasize ageing in place (AIP) as a way to control costs associated with population ageing (1). The Netherlands is the highest spender of long-term care per gross domestic product in the world(2). Most Dutch long-term care services are paid by the national health insurance, with a low percentage of co-payments (3). To cope with the rising long-term care costs, the Netherlands reformed its long-term care policy in 2015 (4). The reform increased informal care duties and encouraged older adults to AIP (4, 5). A substantial component of the Dutch long-term care costs is attributed to dementia care, as it is the most costly disease encompassing 10.6% of the total Dutch healthcare budget (6). The dementia population is known for its requirement of complex and costly care (7). However, it remains unknown how AIP has affected this population. In the years after the reform, preliminary results indicate an increased need for support with crisis situations such as, unplanned hospitalizations, acute nursing home admissions, and hospital deaths (8, 9). These crises situations are not only costly, but can also compromise the safety and well-being of persons with dementia AIP, in addition to the informal caregivers expected to carry the load.

Despite the need for evidenced-based policy to ensure proper healthcare services, people with dementia and informal caregivers are frequently excluded from policy creation; therefore, services often do not fit with their needs or are not provided at the right time (10, 11). To support successful AIP, identifying the care preferences of persons with dementia and informal caregivers is crucial. Whilst identifying these preferences, special attention should be given to persons with dementia and informal caregivers with a migration background, who are known to have a higher risk of developing dementia (12, 13). Despite their high risk, persons with a migration background are underrepresented in research and care (14). It is crucial that persons with a migration background be included in research so healthcare package evidence is representative of the dementia population. To provide recommendations for healthcare packages it is subsequently needed to quantify the identified care preferences, which can be realized with a discrete choice experiment (DCE).

DCEs are a popular stated preference method used to elicit patients' preferences in healthcare on a large scale (15-17). In a DCE participants are presented with a series of alternative hypothetical scenarios. Participants are repetitively asked to select in each alternative hypothetical scenario their preferred option from amongst a presented set of options, e.g., care packages (16). These choice options are characterized by their attributes and corresponding attribute levels (15, 16). An example of an attribute could be emotional support, described as the possibility to talk to someone about personal feelings or concerns. The corresponding attribute levels could be *(1)* peer support group, *(2)* psychologist or *(3)* telephone helpline. The outcomes (choices) from DCEs are analyzed based on the assumption that participants act in a utility maximizing manner, choosing their most preferred option based on the relative overall attractiveness of the included attributes and attribute levels (16, 18). This protocol paper will outline the development of a Dutch national study to create a healthcare package design methodology, built on DCEs, that is person-centered and helps support both informal caregivers and persons with dementia to successfully AIP. Qualitative methods will be used to identify the care needs and preferences of persons with dementia and their informal caregivers. The results of the qualitative studies will provide insights into the attributes (i.e., care components) that will be included in the DCE. For the DCE, a subpopulation analysis of persons with dementia with a migration background is planned.

Aim

The aim of this study is to identify individual and joint preferences of persons with dementia and informal caregivers for in-home support that enables AIP. We also aim to create optimal healthcare packages for persons with dementia and informal caregivers to AIP. For this second aim, choice model inferences will be the basis for creating the most preferred care packages and testing the uptake in a subsample of participants. This will validate the national model inferences and provide policymakers with high quality, understandable and implementable evidence.

METHODS

Overview of the discrete choice experiment

In this protocol paper, the different phases of designing an inclusive DCE study in which persons with dementia and their informal caregivers can participate will be outlined. The study will be conducted between April 2022 and May 2025. Figure 1 illustrates that the first phase is focused on attribute development, in which semi-structured interviews are used to identify important components of care for persons with dementia and informal caregivers AIP. In the second phase persons with dementia and informal caregivers are asked which of the care components are most important to them, leading to a prioritization of the attributes and the development of attribute levels. For phases one and two, the COREQ guidelines for reporting qualitative research will be followed to ensure important aspects of the research team, reflexivity, study design, findings, and analysis are reported (19). The third phase consists of a pilot which is used to test if the content of the DCE is understandable for persons with dementia and informal caregivers, and if the list of included attributes and corresponding attribute levels is complete. Finally, in phase four the researchers conduct the DCE study. An expert panel will be consulted during all the different phases of the study to reflect upon the research design and findings. Phases three and four will be reported according to the STROBE guidelines, developed as a tool for authors to ensure high-quality reporting of observational studies(20). For constructing the DCE design the ISPOR good research practices reports will also be followed (21, 22).

Patient and public involvement

The DCE, including the framing of the questions, will be developed after focus group discussion and multiple individual interviews with persons with dementia and informal caregivers. Persons with dementia, informal caregivers, and policymakers are involved from the start of the study to ensure the appropriateness of the methodology and to help come to meaningful and implementable results (23). Study materials, such as interview guides, will be discussed with persons with dementia and informal caregivers before the start of the study to ensure appropriateness and make necessary changes. Prior to the DCE, person with dementia, informal caregivers, and patient advocacy groups will be asked to voice their opinion about the final list of attributes and corresponding levels included in the DCE. Patient advocacy groups, persons with dementia and informal caregivers are to be included to make sure that the study properly represents these groups, and the study results reflect their needs and preferences. The patient advocacy groups and policymakers are consulted through an expert panel, details of which can be found in the next section. The GRIPP2 checklist will be followed to report the patient and public involvement (24).

The DCE study will identify individual and joint preferences to AIP, allowing for this concept to be implemented elsewhere. The findings of this study will be published in peer-reviewed journals and an easy-to-understand summary of the results will be available through patient organizations such as Alzheimer Nederland and Pharos. Furthermore, the researchers will organize informative events to communicate the study results to persons with dementia and informal caregivers. These events will

For peer review only

be organized together with the patient advocacy groups and informal caregivers. Finally, a policy brief will be written to ensure that the study results are known to policy creators and can help to improve healthcare package design for older adults and informal caregivers.

Expert panel
Throughout the entire study, an expert panel will be consulted on the design and content of the DCE, and on the inferences made from its execution. The expert panel will include researchers who have experience with doing DCEs, policymakers, health insurers, patient advocacy groups, persons with dementia and informal caregivers. The experienced researchers will be asked to provide guidance and feedback on the methodological choices made in the DCE design. Subsequently, the envisioned outcome of the DCE study will be discussed with policymakers and health insurers to gain insights concerning the comprehension and appropriateness of this envisioned outcome. The inclusion of policymakers and health insurers in this phase will allow for the opportunity to make changes to the study design that help to realize implementation.

Participants
This study aims to identify preferences of persons with dementia and informal caregivers aging in place, therefore persons with dementia who live in a long-term care facility will not be included in this study. Additional Exclusion criteria will consist of (a) persons who are cognitively impaired to the extent that no conversation can be held with them; (b) people who are unable to provide informed consent. It is know that dementia diagnoses are often delayed, especially in migration populations (25), therefore people without a formal diagnoses will not be excluded a-priori. However, recruiting participants trough healthcare professionals, day-care centers and social organizations that serve persons with dementia and informal caregivers will ensure that all persons have a dementia indication. For the final DCE, the telephone-based interview for cognitive screening (TICS) will be administered to describe the study participants (26).

Prior to the interviews, focus groups, or DCE choice tasks, the researchers will go through the information letter and informed consent form together with the participants to ensure proper understanding and provide the opportunity to ask for clarifications. Subsequently, participants will be asked to provide written informed consent. If participants are unable to write, verbal continued informed consent will be used (27). Throughout the study, participants will be continuously reminded that they are participating in research to ensure that they remain informed and are comfortable to continue (27). It will be stressed that participation can be stopped at any moment.

Attribute and level development for Dutch persons with dementia and informal caregivers
Study phases one and two are already partly completed, as interviews and focus groups were held with persons with dementia and informal caregivers across the Netherlands to identify attributes and levels (Table one)(28, 29). The identified attributes and levels were similar for persons with dementia and informal caregivers, however they prioritized the care components differently (29). People with dementia prioritized day-to-day help and social care, while for informal caregivers, priorities were information about dementia, organisation of care, and emotional support (29). Unfortunately, no persons with a migration background were included in these qualitative studies. Therefore, the interviews and focus groups will be repeated with persons with dementia and informal caregivers with a migration background to ensure the attributes and levels are applicable to this part of society as they have a higher risk for dementia compared to people with non-migration background (12).

For peer review only

Table 1. Attribute description and corresponding levels		
**Attribute**	**Description**	**Possible levels**
In-home care	I can get assistance at home with personal care such as showering, dressing, or medication	- Daily on a fixed time<br>- 24/7 on demand
Help with daily activities	I can get assistance at home with household tasks such as groceries, laundry, cooking, cleaning, or help with doing my finances	- Once per week<br>- Multiple times per week
Social activities	I can participate in social activities that I like to do	- At a day-care facility<br>- At home
Emotional support	I can talk to someone when I feel down or want to share my worries	- Peer support group<br>- Psychologist<br>- Telephone helpline<br>- Case manager
Information about dementia	I can get information about having dementia	- Telephone helpline<br>- Case manager
Navigating the healthcare system	I can get assistance with organizing care, and help with insurances	- Telephone helpline<br>- Case manager
Home adaptations and tools	I can get home adaptations and tools such as a stair lift, grips in shower and toilet, or a personal alarm	- No reimbursements<br>- Fully reimbursed

Note: current list of attributes may need to be updated after expert panel review and interviews with persons with dementia and informal caregivers with a migration background.

Attribute identification for persons with dementia and informal caregivers with a migration background

To identify care preferences and needs of persons with dementia and informal caregivers with a migration background, semi-structured interviews will be performed across the Netherlands. Participants will primarily include persons with dementia and informal caregivers with a Turkish, Moroccan, or Surinamese background as these are the largest groups of non-western immigrants in the Netherlands and they are known to have a high risk for developing dementia (12). People living with dementia who do not speak Dutch as their first language will be welcome to participate.

Participants will be recruited with the help of day-care centers, general practitioners, and organizations that serve persons with a migration background (Pharos and NOOM). The organizations will inform potential participants about the study, and interested participants will be provided with a bilingual invitation letter. The interviews will be conducted in the preferred language of the participants: Turkish, Moroccan-Arabic, Tarifit, or Dutch (12). Interviews will be conducted by trained bilingual interviewers. Persons with dementia and informal caregivers will be interviewed separately. However, since it is important that participants feel comfortable, if they strongly wish to be interviewed together this will be permitted.

The interviews will be semi-structured, which means that an interview guide (supplementary file 1) will be used to provide some structure, whilst leaving room for new topics to be brought up by the participants. The interview guide will generally be based on the guide (supplementary file 2) of the interview study for persons with non-migration backgrounds (28), with additions based on findings of previous studies that focused on persons with dementia and informal caregivers with a migration background (30, 31). The cultural sensitivity and understandability of the interview guide will be assessed by Pharos, center of expertise in health disparities, and experienced researchers (CS and ÖUB). The interviews will be transcribed and translated by the bilingual interviewer who conducted

For peer review only

the interviews. Respondents will have the opportunity to review the translated transcripts, which will help to ensure proper interpretation and provide opportunities to give clarifications. It will be stressed that this is voluntary, participants are free to choose if they wish to read the translated transcripts. Interviews will be held until data saturation is reached, which is expected after approximately 10 interviews for each group (32). Reflexive thematic analysis will be used to analyze and identify relevant patterns within the data (33, 34). Coding of the data will be done with both a deductive and inductive approach, starting with certain theoretical or empirical assumptions, with an open approach (33). The results will provide a list of attributes (care components) that are important for persons with dementia and informal caregivers with a migration background.

Attribute prioritization and the development of attribute levels

To understand the relative importance of the care components that are identified, mixed focus group sessions will be held with a new sample of persons with dementia and informal caregivers with a Turkish, Moroccan, or Surinamese background (12). Focus groups create an environment in which participants can share their experiences and build on each other's knowledge (29). To ensure a free discussion, informal caregivers will not be participating in the same focus group as their person with dementia (29). Participants will be recruited with the help of previously mentioned organizations. To facilitate dialogue between the participants, focus groups will include participants with similar migration backgrounds and linguistic preferences. The focus groups will be conducted by trained bicultural interviewers. The focus group session will include a quantitative ranking exercise in which persons with dementia and informal caregivers will be asked to list the care components from most important to least important (29). For this ranking exercise, cards with the previously identified care characteristics, containing visual and written information will be used. During the focus group, the reasoning behind the choices made in the ranking exercise will be discussed.

Focus groups will be held until saturation is reached, which is expected after approximately six sessions, each including persons with dementia (n = 4) and informal caregivers (n = 4). Focus groups will be transcribed and translated by the trained bilingual interviewers. Participants will be provided with the opportunity to read the translated transcripts and, if necessary, provide clarifications. Reflexive thematic analysis will be used to analyze and identify relevant patterns within the data (33, 34). Coding of the data will be done with both a deductive and inductive approach, starting with certain theoretical or empirical assumptions, with an open approach (33). The results will consist of a ranking of the attributes (care components) and a description of the attribute levels (characteristics of care components).

Discrete choice experiment pilot

For the DCE pilot the list of attributes that has previously been identified (Table 1) will be updated with the findings of the qualitative studies with persons with dementia and informal caregivers with a migration background. The pilot will include both individual DCE rounds with the persons with dementia (n=4) and informal caregivers (n=4), and dyadic rounds with both the person with dementia and their informal caregiver. The in-person pilot will include the testing of respondents' understanding of choice tasks and the appropriateness of the included attributes and attribute levels. Furthermore, the pilot will help to illustrate if the length of the DCE survey is acceptable. Based on the pilot, changes might need to be made to the DCE survey to increase appropriateness and understandability, as well as manage survey complexity for persons with dementia.

For peer review only

The final discrete choice experiment

The final DCE will include three rounds: (1) persons with dementia, (2) informal caregivers, and (3) dyadic rounds with both the persons with dementia and informal caregivers. The first two rounds will help to elucidate individual preferences, whilst the dyadic round will help to elucidate joint preferences. Identifying joint preferences is important since decisions about care are often made by multiple individuals (35-37). Furthermore, the dyadic DCE round can pose as an opportunity for the person with dementia and their informal caregiver to have an in-depth conversation about their preferences and needs, which could lead to better mutual understanding (38). It is likely that preference adaptation will occur, however as shown in previous research this adaptation can be from both the person with dementia and the informal caregiver (38). Informal caregivers were not primarily dominant in dyadic DCE rounds, in fact the informal caregivers were found to be helpful in assisting the person with dementia to complete more choice tasks (38). An active role of the researcher administering the DCE choice tasks is needed to help guide the dialogue in the joint DCE rounds. People with dementia will be encouraged to state their preference and motivation first to minimize agency of informal caregivers (38).

The complexity of a DCE can be challenging for persons with dementia (39-41). However, a recent study found that persons with moderate cognitive impairment can complete DCE choice tasks (38). Therefore, persons will not be excluded a-priori based on their level of cognitive impairment. The following options in the DCE design will help to lower the cognitive burden for persons with dementia. First, illustrations will be used to visualize the choice sets and included attributes. Figure 2 provides an example of such an illustration (38). The illustrations are made by a graphic designer who has experience illustrating pictures for persons with cognitive impairments. Second, the DCE will have binary choice tasks, asking participants to choose between packages A and B, instead of including multiple alternatives (e.g., additional packages C or D). Participant will complete a maximum of six choice tasks, consisting of three attributes and two corresponding levels (38). Blocked fractional factorial designs (42) will be created with the use of Ngene. Finally, the DCE will start with a practice round to test decision making skills (38). Persons with dementia will be asked to think aloud whilst completing the choice tasks. The think-aloud technique is commonly used in research to make thought processes of participants observable for researchers (43). It has been found useful in supporting persons with dementia to complete DCE choice tasks, as it enables researchers to observe difficulties and, if needed, provide guidance or remind persons with dementia about the rules of the choice tasks (38).

Power calculation

Envisioning a two-alternative choice task and four replicates per dyad, a minimum sample size N≥576 is recommended for a discrete choice experiment to reliably achieve 95% confidence intervals on true uptake probabilities of 0.4 (44). Therefore, the goal will be to achieve a sample size of 600 dyads of persons with dementia and informal caregivers, covering all targeted groups. Purposive sampling of minority groups, people living in urban and rural areas, and people with different educational levels will be used. In addition, sampling across the country will help to make the healthcare package as nationally representative as possible (45).

Participants will be recruited through general practitioners, geriatricians, day-care facilities, and organizations that serve persons with dementia and informal caregivers (Alzheimer Nederland, Pharos, NOOM). The professionals will inform potential participants about the study, and provide them with the (bilingual) information letter. With the participants' consent, the researchers will obtain their

For peer review only

contact information form the professionals. The researchers will contact the potential participant by telephone to answer any remaining questions and to administer the TICS (26). The researchers will schedule an appointment at the participants home to complete the DCE choice tasks in person.

Outcomes

The main outcome of the DCE study will be the relative importance of the attributes (care components). The relative importance of each attribute will be calculated as the difference between the preference weights of the most and least preferred level of that attribute (46). The relative importance will be scaled so that 10 indicates the most preferred attribute. The difference between individual and joint DCE round will be analysed with summary statistics and bivariate comparisons.

A contingent valuation approach will be used to identify the participants' willingness to pay (WTP) for the most preferred programs outside of the DCE (47, 48). While out-of-pockets costs for social care does not currently occur in the Netherlands yet, this may soon change. Therefore, an indication of the WTP is helpful for policy makers and health insurers. At the end of the DCE, participants indicate their preferred envisioned package over their current service provision, they will be asked if they are willing to pay an additional monthly out-of-pocket fee of €50 for this package. If participants answer this question with yes, additional questions with out-of-pocket fees of €100 and €200 will be asked (47). If participants answered no to the initial question of €50, similar questions with an amount of €25 and €12,50 will be asked (47). Finally, participants will be asked to indicate their maximum WTP in an open-ended question (47). For the analysis, the maximum price expressed in the open-ended question will be used and summarized through the median and interquartile range (47).

Statistical analysis

The DCE data will be analysed with a random parameters logit (RPL) model (46). For each attribute level, coefficient estimates will be estimated from the RPL model, which can be interpreted as the relative preference weight (46). Individual and joint models will be created to evaluate preferences of persons with dementia and informal caregivers at the individual and joint DCE rounds.

To analyse if persons with dementia and informal caregivers with a migration background have different preferences, post hoc subgroup analysis will be conducted. Dummy coded variables will be added to the RPL model to identify participants that are part of given subgroups. The attribute levels in the RPL model will be interacted with the dummy variable, and all interaction terms will be added to the original RPL model (46). The estimated parameters of the interaction terms will illustrate the difference in preferences between the subgroup (persons with a migration background) and the reference group (persons without a migration background)(46). This method can also be used to analyse if there are different preferences based on other subgroups, such as socio-economic status.

Finally, the attributes with the highest relative importance can be used to build most preferred programs (healthcare packages). To ensure validity of these results for health insurers and government, the uptake of the most preferred programs will be tested on a subsample of the target population. Model predictions will be verified by creating the most preferred programs according to the preferences of persons with dementia and informal caregivers that were found in the DCE. A new sample will be collected of 50-60 participants to test the uptake of the most preferred programs by asking participants which one they would rather have over their current service provision (49).

DISCUSSION

The discrete choice methodology described in this paper enables persons with dementia and informal caregivers to participate in policy formulation and evaluation. It is assumed that this will help to avoid

crisis situations and improve the quality of life of persons with dementia and their informal caregivers. Furthermore, the outcome of the final DCE will be a ranking of the most preferred care components represented in a care package. This helps health insurers and the ministry of health to maximize their chances for designing healthcare packages that are appropriate, useful, and meaningful.

Furthermore, this study protocol provides an important methodological contribution. It is a clear guide on how to build an inclusive DCE, covering the important methodological choices throughout the required study phases. Moreover, it provides guidelines on how to include underrepresented groups in DCEs. The methodological choices described to help include persons with dementia with a migration background, can be used to include other underrepresented groups such as people with low (health) literacy (50, 51). Finally, this study provides an example of how to overcome language barriers in research and knowledge dissemination.

Strengths and limitations of this study

A strength of this study will be that it is the first study to include informal caregivers and persons with dementia to identify AIP preferences that has been co-designed with health insurers, policymakers, patient advocacy groups, healthcare professionals, researchers, informal caregivers, and persons with dementia. Innovative and rigorous economic methods will be employed to evaluate AIP preferences. Another strength is the inclusion of persons with dementia with different migration backgrounds, which can lead to a better understanding of their needs since they are typically underrepresented in research.

A limitation of this study is the generalizability of results, as not all migrant groups living in the Netherlands may be included. In addition, the chosen recruitment strategies make it unlikely for persons who do not have a diagnoses to be included in this study. Persons with communication difficulties, such as hearing or vision loss will have more difficulties to participate in DCEs. However, the participants ability to complete the DCE will be assessed with practice choice tasks. Finally, enabling persons to participate from their own homes helps to include persons with limited mobility. The research team strives to make the study as inclusive as possible.

Ethics and dissemination

Based on the study protocol, the Ethics Committee (METC) of the Amsterdam University Medical Centre waived the obligation for the study to undergo formal ethical approval as is described under Dutch law in the Medical Research in Humans Act, January 2019 (ref W23_112 #23.137).

Consent

This is a prospective study and pseudonymised data is used, written and continued informed consent will be obtained from the participants prior to participation. This is consistent with current European legislation under the General Data Protection Regulation (GDPR). This study will abide by the Declaration of Helsinki and present ethical requirements.

Authors' contributions

JMV conceptualized the study and developed the study design together with the other researchers. IV and JW contributed to the development of the study design, data collection and analysis. The authors provided input for the study design from their own area of expertise; inclusive research (CS & ÖUB), qualitative research (NL), discrete choice experiments (EBG & JS). IV wrote the manuscript, the co-authors read, edited, and approved the final version.

For peer review only

Funding statement
This work was supported by Alzheimer Nederland, (WE,06-2021-04). In addition, grant support was from The Netherlands Organisation for Scientific Research (https://www.nwo.nl/en) (NWO-Talent-Scheme-Vidi-Grant No. 09150171910002) to EBG.

Competing interest statement
No competing interests to report.

Figure legends
Figure 1. Flowchart study design
Caption: Figure 1: Flowchart study design to create healthcare packages based on discrete choice experiments
Figure 2: Illustrated DCE choice tasks
Caption: Figure 2: Example of an illustrated DCE choice task

References

1. Spasova S, Baeten, R., Coster, S., Ghailani, D., Peña-Casas, R. and Vanhercke, B. Challenges in long-term care in Europe. A study of national policies. Brussels: European Commission, (ESPN) ESPN; 2018.
2. Organisation for Economic Cooperation and Development. long-term care spendings and unit costs Paris: OECD publishing 2021.
3. Bakx P, Schut E, Wouterse B. Price setting in long-term care in the Netherlands. Erasmus School of Health Policy and Management, November. 2020.
4. Alders P, Schut FT. The 2015 long-term care reform in the Netherlands: Getting the financial incentives right? Health Policy. 2019;123(3):312-6.
5. Maarse JH, Jeurissen PP. The policy and politics of the 2015 long-term care reform in the Netherlands. Health Policy. 2016;120(3):241-5.
6. RIVM. Effecten van vergrijzing steeds beter zichtbaar in uitgaven gezondheidszorg 2022 [Available from: https://www.rivm.nl/nieuws/effecten-van-vergrijzing-steeds-beter-zichtbaar-in-uitgaven-gezondheidszorg#:~:text=In%202019%20werd%20in%20Nederland,naar%20de%20kosten%20van%20ziekten.
7. Wimo A, Guerchet M, Ali G-C, Wu Y-T, Prina AM, Winblad B, et al. The worldwide costs of dementia 2015 and comparisons with 2010. Alzheimer's & Dementia. 2017;13(1):1-7.
8. Francke A, Heide I, Bruin Sd, Gijsen R, Poos R, Verbeek M, et al. Een samenhangend beeld van dementie en dementiezorg: kerncijfers, behoeften, aanbod en impact. Themarapportage van de Staat van Volksgezondheid en Zorg. 2018.
9. MacNeil-Vroomen JL, van der Steen JT, Holman R, Monin JK, Buurman BM. Hospital deaths increased after reforms regardless of dementia status: an interrupted time-series analysis. Journal of the American Medical Directors Association. 2021;22(7):1507-11.
10. Van der Roest HG, Meiland FJ, Comijs HC, Derksen E, Jansen AP, Van Hout HP, et al. What do community-dwelling people with dementia need? A survey of those who are known to care and welfare services. International psychogeriatrics. 2009;21(5):949-65.
11. Van Der Roest HG, Meiland FJ, Maroccini R, Comijs HC, Jonker C, Dröes R-M. Subjective needs of people with dementia: a review of the literature. International Psychogeriatrics. 2007;19(3):559-92.
12. Parlevliet JL, Uysal-Bozkir Ö, Goudsmit M, van Campen JP, Kok RM, Ter Riet G, et al. Prevalence of mild cognitive impairment and dementia in older non-western immigrants in the Netherlands: a cross-sectional study. Int J Geriatr Psychiatry. 2016;31(9):1040-9.

For peer review only

13. Selten J-P, Termorshuizen F, van Sonsbeek M, Bogers J, Schmand B. Migration and dementia: a meta-analysis of epidemiological studies in Europe. Psychological Medicine. 2021;51(11):1838-45.
14. Schmachtenberg T, Monsees J, Hoffmann W, van den Berg N, Stentzel U, Thyrian JR. Comparing national dementia plans and strategies in Europe–is there a focus of care for people with dementia from a migration background? BMC Public Health. 2020;20:1-10.
15. de Bekker-Grob EW, Ryan M, Gerard K. Discrete choice experiments in health economics: a review of the literature. Health economics. 2012;21(2):145-72.
16. Soekhai V, de Bekker-Grob EW, Ellis AR, Vass CM. Discrete choice experiments in health economics: past, present and future. Pharmacoeconomics. 2019;37(2):201-26.
17. Friedel JE, Foreman AM, Wirth O. An introduction to "discrete choice experiments" for behavior analysts. Behavioural processes. 2022;198:104628.
18. Clark MD, Determann D, Petrou S, Moro D, de Bekker-Grob EW. Discrete choice experiments in health economics: a review of the literature. Pharmacoeconomics. 2014;32(9):883-902.
19. Tong A, Sainsbury P, Craig J. Consolidated criteria for reporting qualitative research (COREQ): a 32-item checklist for interviews and focus groups. International journal for quality in health care. 2007;19(6):349-57.
20. Cuschieri S. The STROBE guidelines. Saudi J Anaesth. 2019;13(Suppl 1):S31-s4.
21. Johnson FR, Lancsar E, Marshall D, Kilambi V, Mühlbacher A, Regier DA, et al. Constructing experimental designs for discrete-choice experiments: report of the ISPOR conjoint analysis experimental design good research practices task force. Value in health. 2013;16(1):3-13.
22. Bridges JF, de Bekker-Grob EW, Hauber B, Heidenreich S, Janssen E, Bast A, et al. A roadmap for increasing the usefulness and impact of patient-preference studies in decision making in health: A good practices report of an ISPOR task force. Value in Health. 2023;26(2):153-62.
23. Illes J, Lawson A, McDonald PJ. Ethical Considerations for Discrete Choice Experiments with Caregivers. Journal of Empirical Research on Human Research Ethics. 2022;17(4):426-30.
24. Staniszewska S, Brett J, Simera I, Seers K, Mockford C, Goodlad S, et al. GRIPP2 reporting checklists: tools to improve reporting of patient and public involvement in research. BMJ. 2017;358:j3453.
25. Goudsmit M, Uysal-Bozkir Ö, Parlevliet JL, van Campen JP, de Rooij SE, Schmand B. The Cross-Cultural Dementia Screening (CCD): A new neuropsychological screening instrument for dementia in elderly immigrants. Journal of Clinical and Experimental Neuropsychology. 2017;39(2):163-72.
26. Brandt J, Spencer M, Folstein M. The telephone interview for cognitive status. Neuropsychiatry Neuropsychol Behav Neurol. 1988;1(2):111-7.
27. McKeown J, Clarke A, Ingleton C, Repper J. Actively involving people with dementia in qualitative research. Journal of clinical nursing. 2010;19(13-14):1935-43.
28. Vullings I, Labrie N, Wammes JD, de Bekker-Grob EW, MacNeil-Vroomen J. Important components for Dutch in-home care based on qualitative interviews with persons with dementia and informal caregivers. Health Expectations. 2020;23(6):1412-9.
29. Wammes JD, Labrie NH, Agogo GO, Monin JK, de Bekker-Grob EW, MacNeil Vroomen JL. Persons with dementia and informal caregivers prioritizing care: A mixed-methods study. Alzheimer's & Dementia: Translational Research & Clinical Interventions. 2021;7(1):e12193.
30. Lillekroken D, Halvorsrud L, Gulestø R, Bjørge H. Family caregivers' experiences of providing care for family members from minority ethnic groups living with dementia: A qualitative systematic review. Journal of Clinical Nursing. 2021.
31. Duran-Kiraç G, Uysal-Bozkir Ö, Uittenbroek R, van Hout H, Broese van Groenou MI. Accessibility of health care experienced by persons with dementia from ethnic minority groups and formal and informal caregivers: A scoping review of European literature. Dementia. 2022;21(2):677-700.
32. Hennink MM, Kaiser BN, Marconi VC. Code saturation versus meaning saturation: how many interviews are enough? Qualitative health research. 2017;27(4):591-608.
33. Braun V, Clarke V. Using thematic analysis in psychology. Qualitative research in psychology. 2006;3(2):77-101.

For peer review only

34. Braun V, Clarke V. Reflecting on reflexive thematic analysis. Qualitative research in sport, exercise and health. 2019;11(4):589-97.
35. Corfman KP, Lehmann DR. Models of cooperative group decision-making and relative influence: An experimental investigation of family purchase decisions. Journal of Consumer Research. 1987;14(1):1-13.
36. Hensher DA, Beck MJ, Rose JM. Accounting for preference and scale heterogeneity in establishing whether it matters who is interviewed to reveal household automobile purchase preferences. Environmental and Resource Economics. 2011;49(1):1-22.
37. Groen-van de Ven L, Smits C, Span M, Jukema J, Coppoolse K, de Lange J, et al. The challenges of shared decision making in dementia care networks. International Psychogeriatrics. 2018;30(6):843-57.
38. Wammes JD, Swait JD, de Bekker-Grob EW, Monin JK, Labrie NH, MacNeil Vroomen JL. Dyadic Discrete Choice Experiments Enable Persons with Dementia and Informal Caregivers to Participate in Health Care Decision Making: A Mixed Methods Study. Journal of Alzheimer's Disease. 2022(Preprint):1-10.
39. Chester H, Clarkson P, Davies L, Sutcliffe C, Davies S, Feast A, et al. People with dementia and carer preferences for home support services in early-stage dementia. Aging & Mental Health. 2018;22(2):270-9.
40. O'Philbin L, Woods B, Holmes E. People with dementia and caregiver preferences for digital life story work service interventions. A discrete choice experiment and digital survey. Aging & Mental Health. 2020;24(2):353-61.
41. Ratcliffe J, Hutchinson C, Milte R, Nguyen KH, Welch A, Caporale T, et al. How do people with dementia and family carers value dementia-specific quality of life states? An explorative "Think Aloud" study. Australasian Journal on Ageing. 2019;38:75-82.
42. Jaynes J, Wong WK, Xu H. Using blocked fractional factorial designs to construct discrete choice experiments for healthcare studies. Statistics in medicine. 2016;35(15):2543-60.
43. Uysal-Bozkir Ö, Fokkema T, MacNeil-Vroomen JL, van Tilburg TG, de Rooij SE. Translation and validation of the De Jong Gierveld loneliness scale among older migrants living in the Netherlands. The Journals of Gerontology: Series B. 2017;72(1):109-19.
44. Louviere JJ, Hensher DA, Swait JD. Stated choice methods: analysis and applications: Cambridge university press; 2000.
45. Valliant R, Dever JA, Kreuter F. Practical tools for designing and weighting survey samples: Springer; 2013.
46. Rosenow F, Winter Y, Leunikava I, Brunnert M, Joeres L, Sutphin J, et al. Relative importance of clinical outcomes and safety risks of antiseizure medication monotherapy for patients and physicians: Discrete choice experiment eliciting preferences in real-world study" VOTE". Epilepsia. 2022;63(2):451-62.
47. Vo NX, Nguyen TTH, Van Nguyen P, Tran QV, Vo TQ. Using contingent valuation method to estimate adults' willingness to pay for a future coronavirus 2019 vaccination. Value in Health Regional Issues. 2021;24:240-6.
48. Basu R. Willingness-to-pay to prevent Alzheimer's disease: a contingent valuation approach. International journal of health care finance and economics. 2013;13(3):233-45.
49. Janssen EM, Marshall DA, Hauber AB, Bridges JF. Improving the quality of discrete-choice experiments in health: how can we assess validity and reliability? Expert review of pharmacoeconomics & outcomes research. 2017;17(6):531-42.
50. Hughson J-a, Woodward-Kron R, Parker A, Hajek J, Bresin A, Knoch U, et al. A review of approaches to improve participation of culturally and linguistically diverse populations in clinical trials. Trials. 2016;17(1):1-10.
51. Woodward-Kron R, Hughson J-a, Parker A, Bresin A, Hajek J, Knoch U, et al. Culturally and linguistically diverse populations in medical research: perceptions and experiences of older Italians, their families, ethics administrators and researchers. Journal of public health research. 2016;5(1):jphr. 2016.667.

For peer review only

For peer review only

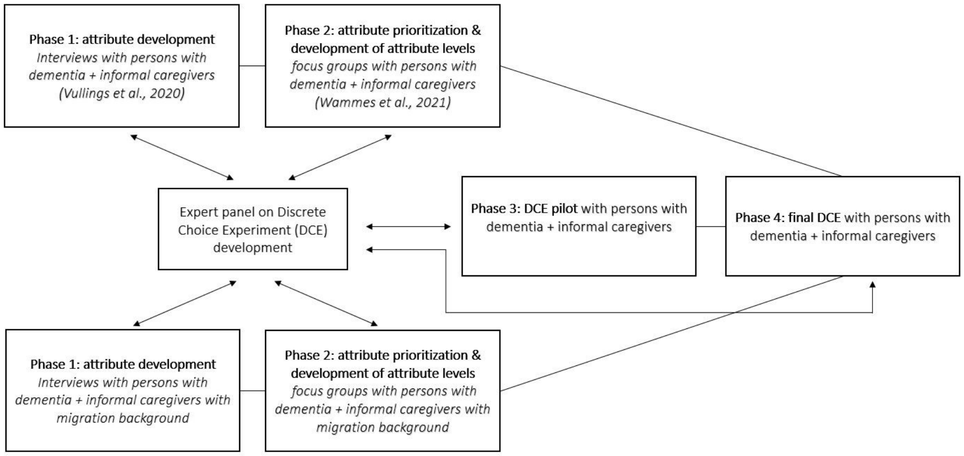

Figure 1: Flowchart study design to create healthcare packages based on discrete choice experiments.

378x184mm (96 x 96 DPI)

	Package A	Package B
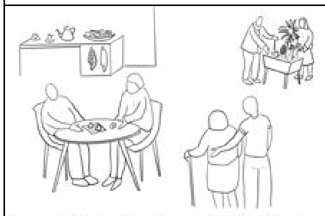 I can participate in activities that I like and socialize with other people	**Once per week** activities are organized **at a daycare center**	**Multiple times per week** there is someone **coming to my house** to do activities together
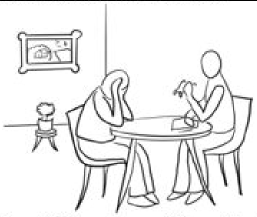 I can talk to someone if I want to share my worries or concerns.	I can talk to a **peer support** group	I can talk to my **case manager**
Select your preference	☐	☐

Figure 2: example of an illustrated DCE choice task

184x114mm (96 x 96 DPI)

Introduction: Thank you for participating in this study. My name is [short introduction interviewer]. The research that we are doing is focused on persons who are living at home. I would like to talk with you about your living situation at home, the daily things that you do and the help that you receive. If I ask a question that is not clear, feel free to let me know. There are no wrong answers, you can say anything that comes to mind. I would like to record the interview, if that is ok with you. The recordings will only be shared with my colleagues. The conversation will not be shared with your doctor or family. We will leave out your name. your name will be replaced by a number. *[reading information letter + informed consent – singing informed consent – start recording]*

Before we start, I have a few general questions that I would like to ask you: 1) What education did you receive? 2) Where were you born? 3) Where were your parents born? 4) How long have you lived in the Netherlands? 5) How old are you? 6) Where do you live? 7) Do you live alone? 8) Where does your family live?

Theme	Explanation theme	Examples of questions
Introduction	Start-up questions giving respondent room to talk and bring up topics.	• Can you tell something about yourself?<br>• How is it going at home?<br>• How is your health?
Daily activities	Talk about: (help with) cleaning, cooking, showering, getting dressed etc.<br><br>Have attention for religious activities praying, going to mosque/church.<br><br>Find out if they get help with these activities, from who, and how they value this help.	• What are daily activities that you do?<br><br>• What does your day look like?
Social activities	Talk about: social activities such as, female/male groups, visiting other persons etc.<br><br>Ask if people go to a daycare facility.<br><br>For informal caregivers: ask if they have time to do things for themselves.	• What are things that you like to do?<br><br>• What are things that you like to do with other people?
Adaptations in the home	Talk about: adaptations such as a stair lift, grips in the shower, alarm system etc.	• What changes are made in your house?<br>• What are things that you would like to change about your house?

For peer review only

Information	Talk about: information received after diagnosis about what to expect or how to cope with dementia.	• Have you gotten information about what to expect (after diagnosis)?<br>• What questions do you have about dementia?
Contact with care professionals	Talk about: how people experience contact with care professionals such as home care, general practitioner, and the case manager dementia.	• What do you think of the home care providers?<br>• How is your contact with the general practitioner?
Navigating the healthcare system	Talk about: does the person arrange care themselves or do they get help (from family, general practitioner, or case manager).<br><br>Do they struggle with this or not.	• How is the care arranged?<br>• Who arranges this support/care?<br>• How would you like to be supported with the arrangement of care?
Emotional support	Talk about: how people feel about their situation, their worries or concerns.<br><br>Do they talk about this and with who.	• How do you feel about your situation (dementia diagnosis)?<br>• do you have someone to discuss your concerns with?
Closing questions	Question to end the interview, ask if participants want to add something.	• What are things that you would like to get help with?<br>• Are there things that you would like to add?

Examples of probing questions

- With who do you do this? / Who helps you with this?
- How often do you do this? / How often do you get this help?
- What do you like about this help/support?
- What do you dislike about this (help/support)?

Thank you so much for sharing your story. *[ask if people wat to be informed about the results of the study].*

For peer review only

Introduction: Thank you for participating in this study. My name is [short introduction interviewer]. The research that we are doing is focused on persons who are living at home. I would like to talk with you about your living situation at home, the daily things that you do and the help that you receive. If I ask a question that is not clear, feel free to let me know. There are no wrong answers, you can say anything that comes to mind. I would like to record the interview, if that is ok with you. The recordings will only be shared with my colleagues. The conversation will not be shared with your doctor or family. We will leave out your name. your name will be replaced by a number. *[reading information letter + informed consent – singing informed consent – start recording]*

Before we start, I have a few general questions that I would like to ask you: 1) What education did you receive? 2) Where were you born? 3) How old are you? 4) Where do you live? 5) Do you live alone? 6) Where does your family live?

Theme	Explanation theme	Examples of questions
Introduction	Start-up questions giving respondent room to talk and bring up topics.	• Can you tell something about yourself?<br>• How is it going at home?<br>• How is your health?
Daily activities	Talk about: (help with) cleaning, cooking, showering, getting dressed etc.<br><br>Find out if they get help with these activities, from who and how they value this help.	• What are daily activities that you do?<br>• What does your day look like?<br><br>• Do you receive help with these daily activities?<br>• How would you like to be helped with these activities?
Social activities	Talk about: social activities such as visiting other persons, playing cards etc.<br><br>Ask if people go to a daycare facility + how they value this.<br><br>For informal caregivers: ask if they have time for themselves, to do their own things.	• What are things that you like to do?<br>• What are things that you like to do with other people?<br><br>• With who do you like to do these activities?<br>• How would you like to be supported in this?
Adaptations in the home	Talk about: adaptations such as a stair lift, grips in the shower, alarm system etc.	• What changes are made in your house?<br>• What are things that you would like to change about your house?

Information	Talk about: information received after diagnosis.<br><br>Did they get information about what to expect or how to cope with dementia.	• Have you gotten information about what to expect (after diagnosis)?<br>• How was the support of your general practitioner/ case manager dementia?<br><br>• What questions do you have about dementia?
Cognitive training	Talk about: if people use tools to help them remember.	• How do your memory problems affect you?<br><br>• What tools do you use to help you remember?
Emotional support	Talk about: how people feel about their situation, their worries or concerns.<br><br>Do they talk about this and with who.	• How do you feel about your situation (dementia diagnosis)?<br><br>• do you have someone to discuss your concerns with?
Closing questions	Question to end the interview, ask if participants want to add something.	• What are things that you would like to get help with?<br><br>• Are there things that you would like to add?

Examples of probing questions

- With who do you do this? / Who helps you with this?
- How often do you do this? / How often do you get this help?
- What do you like about this help/support?
- What do you dislike about this (help/support)?

Thank you so much for sharing your story. *[ask if people wat to be informed about the results of the study].*

For peer review only
